# Substrate Channeling in Compartmentalized Nanoreactors

**DOI:** 10.1021/acs.macromol.4c00697

**Published:** 2024-07-03

**Authors:** Fangbei Liu, Peiyuan Qu, Jeremy Weiss, Kunhao Guo, Marcus Weck

**Affiliations:** Molecular Design Institute and Department of Chemistry, New York University, New York, New York 10003-6688, United States

## Abstract

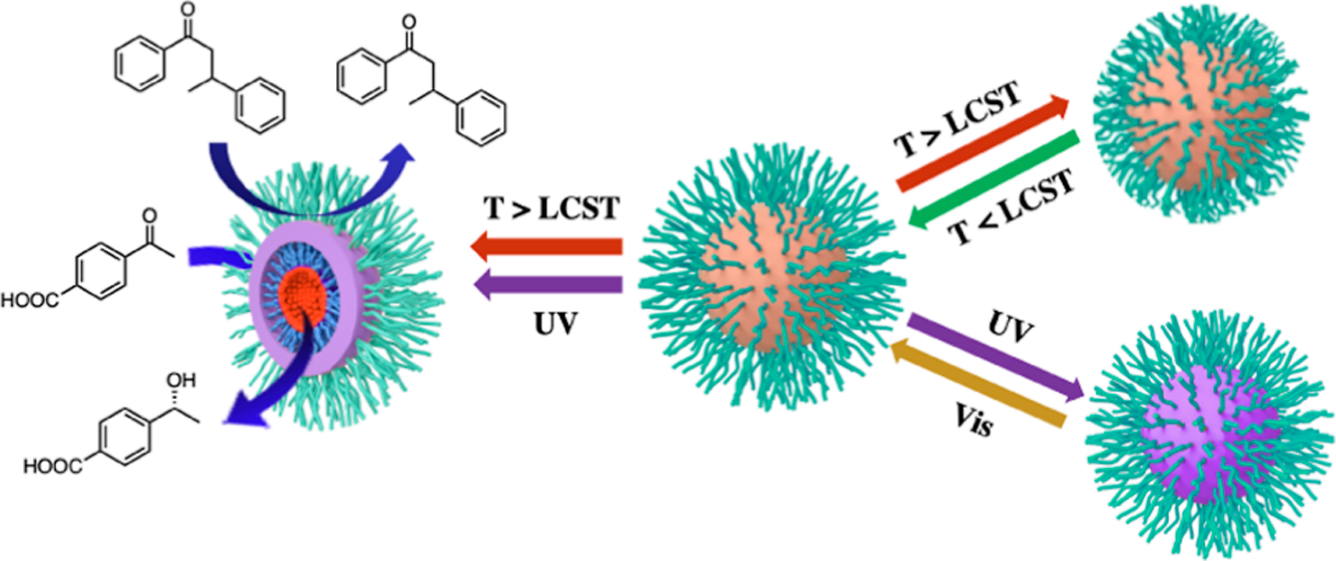

Thermo- and photoresponsive nanoreactors based on shell cross-linked micelles (SCMs) for the rhodium-catalyzed asymmetric transfer hydrogenation (ATH) of ketones have been developed from poly(2-oxazoline) triblock terpolymers. The nanoreactors incorporate thermoresponsive poly(2-isopropyl-2-oxazoline) as the hydrophilic corona and are covalently cross-linked with a photoswitchable spiropyran molecule. UV irradiation or changes in temperature trigger morphology switching of the polymer-based nanoreactors that alters the hydrophobicity in separate layers of the SCMs, resulting in dynamic substrate selectivity of the ATH in water. Control experiments and kinetic studies show that the thermoresponsive outer layer induces the gated behavior for more hydrophobic substrates, whereas the photoresponsive cross-linking layer induces the gated behavior for less hydrophobic substrates. The nanoreactors mimic the multichannels in Nature, transporting substrates and reagents into the catalytic core which can be controlled through external triggers such as temperature and light wavelengths. Additionally, the nanoreactors can be easily recovered and reused with continued high activity and selectivities.

## Introduction

Enzymes catalyze sequential reactions by controlling the mass transport of reactants and intermediates through chemical and physical mechanisms in metabolic pathways.^1^ This exquisite control of intermediates along a pathway is termed substrate channeling.^2^ Channeling promotes the direct transfer of substrates from one active site to another without diffusion to the bulk environment, enabling high yields and selectivities of compounds.^3,4^ Synthetic systems draw inspiration from the nanotunnels found in Nature to fabricate catalytic nanoreactors by aiming to mimic efficient transport and channeling in natural systems. Among these synthetic systems, substrate channeling refers to the controlled transfer of reactants or intermediates between different compartments or components within a system.^5−7^ In this context, channels in artificial nanoreactors are not solely defined as substantial tunnels within the structure; rather, they represent the diffusion pathway through which substrates traverse between compartments. These artificial systems include synthetic liposomes,^8,9^ polymersomes,^10−14^ nanogels,^15−17^ and micellar nanoreactors.^18−22^ Vesicles, including liposomes and polymersomes, offer a distinct advantage by encapsulating both hydrophilic and hydrophobic reactants within their interior and membrane, respectively. However, liposomes always have problem of leaking.^23^ In contrast, polymersomes exhibit higher structural stability and durability, yet they show poor permeability to common ions.^22^ Small organic molecules and even water have difficulty penetrating the bilayer membrane of polymersomes. Nanogels, with their three-dimensional network structure, offer a high-surface area, enabling the efficient encapsulation of active compounds.^24^ Nonetheless, diffusion rates in nanogels can be inhibited by the size of molecules, with larger molecules experiencing slower diffusion through the gel network.^25^ Meanwhile, micellar nanoreactors enable transportation of hydrophobic organic compounds due to the micelle’s hydrophobic inner core. Furthermore, they can effectively regulate the diffusion of substrates based on hydrophobicity and size.^26,27^

In Nature, conformational changes of enzymes control unidirectional channeling, enabling precise substrate diffusion into catalytically active sites.^28,29^ Diffusion of substrates within nanochannels can be controlled using stimuli such as pH,^30,31^ temperature,^32−34^ or light.^35,36^ Nature’s precise control of substrates, reaction progress, and stabilization of intermediates minimizes energy and resources spent on unproductive reaction pathways.^37^ To mimic the controlled channeling in Nature, stimuli-responsive molecules are employed to regulate and control the process of substrate channeling within the artificial nanoreactors.^38−40^ Previously, we introduced photoresponsive molecules to control substrate channeling by generating polymer-based micelles containing photoresponsive cross-linked block terpolymers.^27,41^ These micelles open and close channeling pathways in response to UV/visible light. While synthetic methods have realized substrate channeling in simple systems, imitating Nature’s substrate regulation over multiple channels and regions remains an unfulfilled goal.^42^ Incorporation of two or more independently triggerable units within an artificial nanoreactor to control the reaction pathways has not yet been realized.

**Scheme 1:**
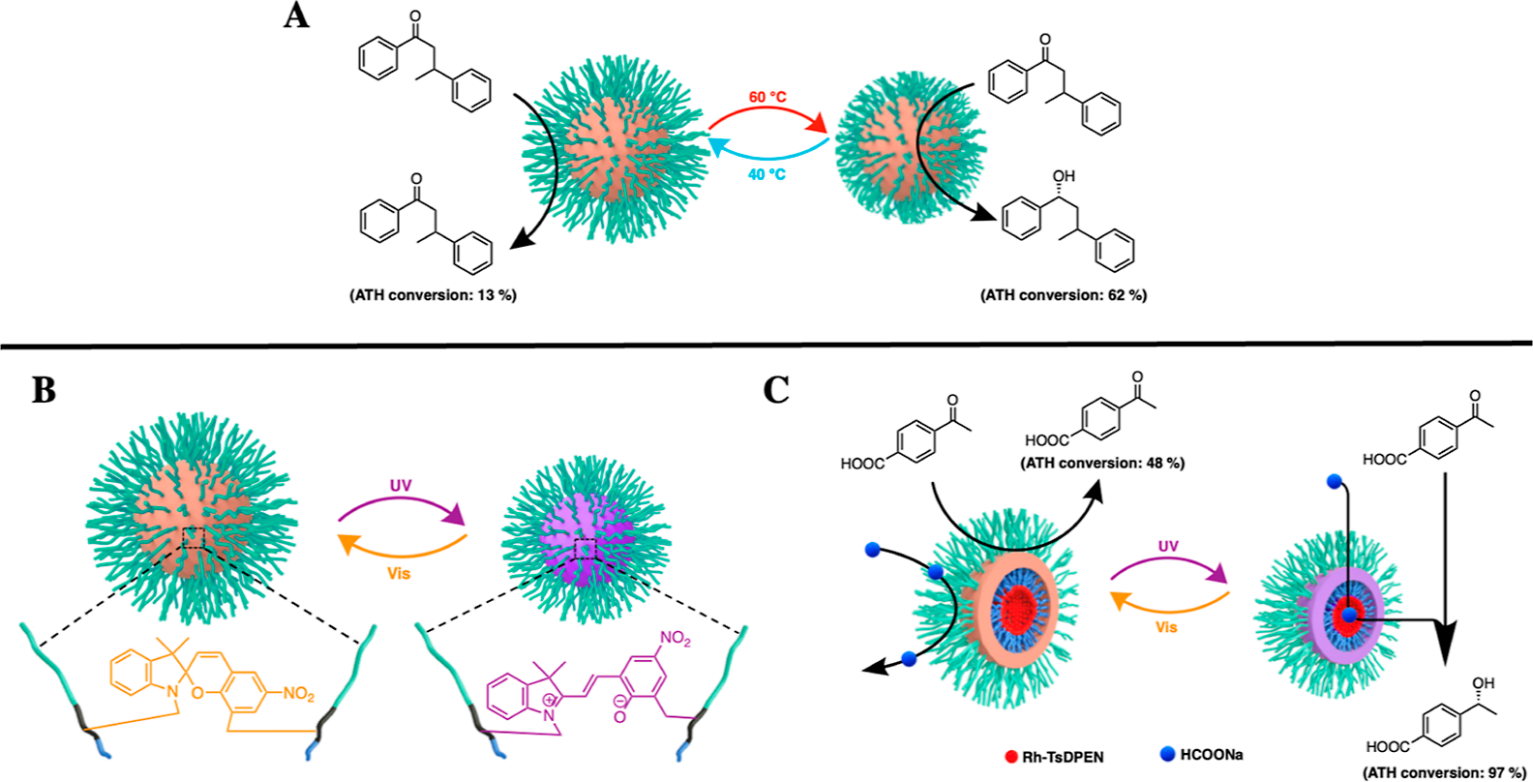
Schematic Representation of the Thermo- and Photo-regulation; (A) Thermoresponsive SCM with Reversible Swelling/Shrinking Behavior at 40 °C/60 °C; Gated Behavior Induced by the Thermoresponsive Outer Layer Is Particularly Effective for More Hydrophobic Substrates; (B) Reversible Photochromism between Spiropyran Cross-Linked SCM and the Zwitterionic Merocyanine Cross-Linked SCM through Vis/UV Light Irradiation; (C) Upon UV Light Irradiation, the Photo-Triggered Transition of the Cross-Linked Layer Enables the Diffusion of HCOONa to the Catalytic Core, Leading to a Higher Conversion of Substrates; Gated Behavior Induced by the Photoresponsive Cross-Linked Layer Is Particularly Effective for Less Hydrophobic Substrates

Amphiphilic block copolymers which self-assemble in solution can form micellar structures with channels.^6,7,43,44^ When polymers are functionalized with responsive units, such as photoswitchable spiropyran, polymeric micelle-supported catalytic systems can change their activity and selectivity, opening and closing channels in response to external stimuli.^45−48^ Shell cross-linked micelles (SCMs), in particular, have the potential to functionalize multiple domains.^18,22,49^ SCMs, composed of a hydrophobic core, a hydrophilic corona, and a cross-linked shell, can be used as nanoreactors for reactions under sustainable conditions (*e.g.*, water as the only solvent). The cross-linked layer stabilizes the micellar structure, resulting in no critical micelle concentration. These materials can be isolated and stored.^50−52^ Additionally, the cross-linked shell layer provides an external shielding preventing metal leaching and allowing catalysts recovery and recycling.^26^ We rationalize that the compartmentalized structure of SCMs will enable substrate channeling by controlling the precise positioning of catalysts and adding responsive units in separate domains. In particular, by incorporating responsive units, the SCMs can undergo structural modifications after external stimuli have been applied, resulting in control of substrate transportation and selectivity, which mimics substrate channeling in Nature. One example of an external stimulus that can be applied is temperature. We hypothesize that poly(2-oxazoline)s (POs)-based SCMs containing small hydrophobic side-chains, such as poly(2-isopropyl-oxazoline) (PiPrOx), exhibit thermoresponsiveness with lower critical solution temperature (LCST) behavior close to body temperature depending on polymer molar mass.^53−56^ Upon heating these POs above their cloud point (*T*_cp_), they should undergo a transition from a hydrophilic coil to a hydrophobic globule thereby opening and/or closing compartments.^57−60^ A similar hydrophobic-to-hydrophilic transition happens when SCMs contain spiropyran, a photochromic molecule.^61,62^ Spiropyran reversibly isomerizes between the hydrophobic spiropyran form and the hydrophilic zwitterionic merocyanine when irradiated with UV light (Scheme 1B).

In this contribution, we report such a system by synthesizing a nanoreactor based on SCM with thermoresponsive PiPrOx as the hydrophilic corona and photoresponsive spiropyran in the cross-linking layer. A rhodium catalyst is immobilized within the hydrophobic core of the SCMs to perform asymmetric transfer hydrogenation (ATH), a powerful method for producing chiral compounds from unsaturated substrates.^63,64^ Temperature and light trigger the hydrophobic–hydrophilic transitions occurring in the corona and the shell of the nanoreactors and, ultimately, enable substrate channeling and regulate substrate selectivity based on hydrophobicity.

## Results and Discussion

**Scheme 2:**
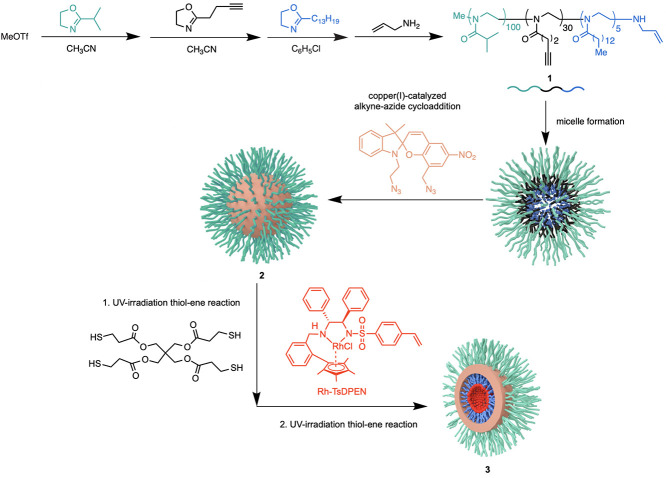
Synthetic Scheme towards the Fabrication of Thermo- and Photoresponsive Nanoreactors

**Figure 1:**
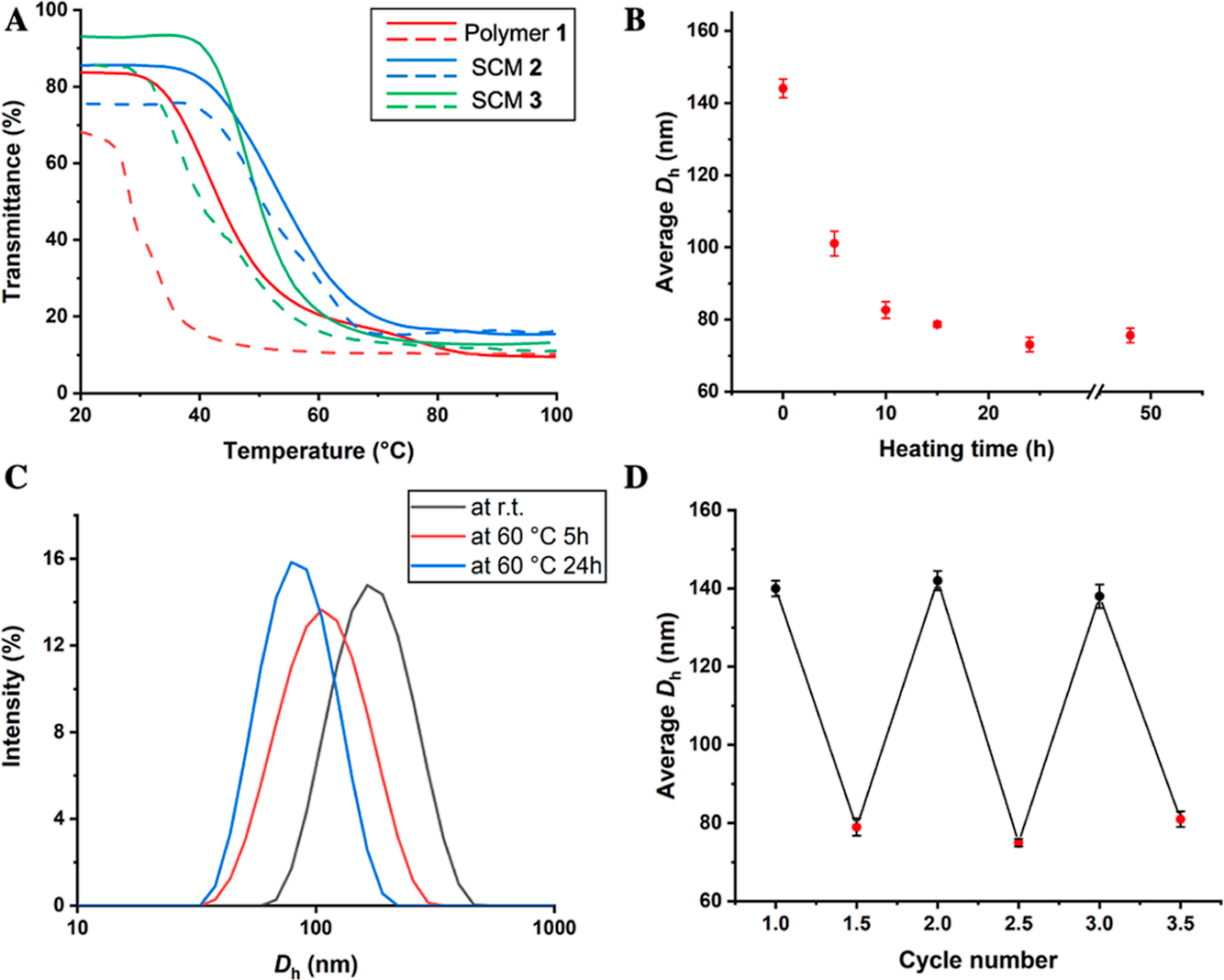
Thermoresponsiveness of the SCMs. (A) Thermal UV/vis spectra of polymer **1** and SCMs **2** and **3**. The solid curves are heating curves, and the dashed curves are cooling curves. Measurement conditions: micelle solution of 1.0 mg/mL in water; heating rate, 1 °C/min; wavelength, 700 nm. (B) At 60 °C, *D*_h_ of SCM **3** at 1.0 mg/mL in water determined by DLS decreases with heating time. (C) DLS traces of SCM **3** at room temperature [polydispersity index (PDI) of 0.177] and at 60 °C heating for 5 h (PDI of 0.126) and 24 h (PDI of 0.102). (D) *D*_h_ of three consecutive heat–cooling cycles determined by DLS. Black dots represent *D*_h_ at room temperature, and red dots are *D*_h_ at 60 °C.

The design strategy of the SCM-based nanoreactors is presented in Scheme 2. Our strategy starts with the synthesis of amphiphilic poly(2-oxazoline)-based ABC triblock terpolymers using living cationic ring-opening polymerization with methyl triflate as the initiator.^65,66^ Triblock terpolymer **1** is designed to include a thermoresponsive PiPrOx hydrophilic block, a middle block containing a terminal alkyne for cross-linking, a hydrophobic block poly(2-tridecanyl-2-oxazoline) with an alkyl group, and a terminal allylamine as a functional handle for postpolymerization modification in the micellar core. Polymerization progress was monitored by ^1^H NMR spectroscopy. Block terpolymers with degrees of polymerization of 100, 30, and 5 for each block, respectively (Figure S1), were obtained. The apparent molecular weight (*M*_n_^app^) and dispersity (*D̵*) as determined by gel-permeation chromatography were 8000 g/mol and 1.30, respectively. The thermoresponsive LCST behavior of polymer **1** was confirmed through turbidimetry measurements by UV/vis spectroscopy (Figure 1A) with low hysteresis between heating and cooling cycles.

Next, we dissolved polymer **1** in water at a concentration of 1.0 mg/mL to form micelles. The micelle nanostructure was characterized by dynamic light scattering (DLS) (Figure S5, left). No nanostructures were observed by DLS in organic solvents such as methanol (Figure S5, right). After micelle formation, copper(I)-catalyzed alkyne–azide cycloaddition was used to cross-link the micelle between the bifunctional azide-containing spiropyran cross-linker and the alkynyl block in water to afford SCM **2**. The covalent cross-linking was confirmed by NMR (Figures S6 and S7) and FT-IR spectroscopies (Figure S8) by following the disappearance of the characteristic alkyne signals at 1.98 ppm in the ^1^H NMR spectrum, 84.7 and 71.0 ppm in the ^13^C NMR spectrum, and 3235.6 cm^–1^ in the FTIR spectrum and the presence of the aromatic spiropyran signal in the ^1^H NMR spectrum and IR spectrum at 800.0 cm^–1^. After cross-linking, the SCM is stable in organic solvents (methanol) (Figure S9), indicating successful cross-linking of the micelles.

Next, we investigated the photoresponsiveness of SCM **2**. SCM **2** was dispersed in water at 1.0 mg/mL and filtered with a 0.45 μm syringe filter. The micelle solution was exposed to visible light (λ = 550 nm) for 15 min followed by UV irradiation (λ = 350 nm) for 15 min. A color change was observed from yellow to dark purple upon switching from visible to UV light exposure. Once the first cycle was completed, the same UV/vis irradiation was repeated for five consecutive cycles. Hydrodynamic diameters (*D*_h_) measured by DLS of SCM **2** fluctuated between 78 and 83 nm under visible light. The *D*_h_ decreased to 70 ± 2 nm upon irradiation with UV light (Figure S10). We hypothesize that the smaller micellar structure under UV light is due to the photoresponsive cross-linker switching to the more hydrophilic merocyanine form (Scheme 1B). This causes the hydrophilic area of SCM **2** to become larger, decreasing the packing parameter and increasing the curvature of the micellar conformation, resulting in the shrinkage of the overall micelle size.^67^ The micelle *D*_h_ change repeated consistently for five UV/vis cycles, indicating the photoresponsive nature of SCM **2**.

The thermoresponsiveness of SCM **2** was confirmed by turbidimetry measurements using UV/vis spectroscopy. The *T*_cp_ of SCM **2** was determined at the midpoint of the heating curves to be 56 °C, which is slightly lower than the *T*_cp_ 58 °C of polymer **1** (Figure 1A). Cross-linking of the polymers increases the hydrophobicity of the micelles, allowing the thermoresponsive corona to push out water at lower temperature.^68^

**Figure 2:**
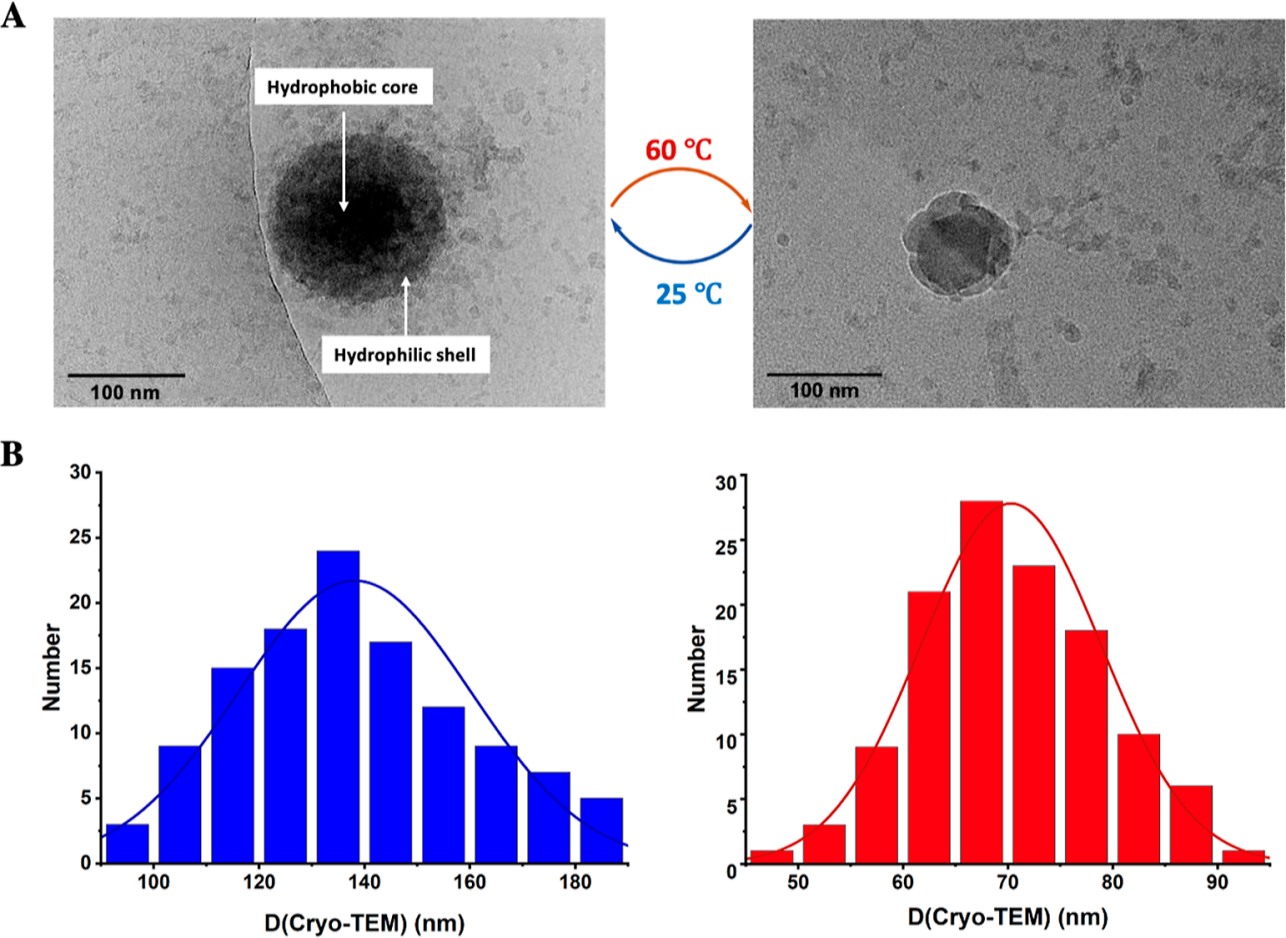
Morphological investigations of SCM **3**. (A) Cryo-TEM images of SCM **3** at 25 °C (left) and 60 °C (right) switching. A core–shell structure was observed. Scare bar = 100 nm. (B) Statistical size distribution of SCM **3** at 25 °C (left) and 60 °C (right) based on 120 micelles from cryo-TEM images.

Next, chiral Rh-TsDPEN was attached to the core of SCM **2** to fabricate SCM **3**. We reacted the alkene functionalities in the core with a multivalent tetrathiol linker through thiol–ene click chemistry (Scheme 2). The ratio of the alkene to tetrathiol linker was 1:1, leaving an average of three free thiols per polymer chain for a second thiol–ene click addition with an alkene-functionalized Rh-TsDPEN. A rhodium loading of 1.1% was determined by inductively coupled plasma mass spectrometry, corresponding to 1.70 Rh-TsDPEN per polymer chain for SCM **3**. The cryogenic-TEM (cryo-TEM) images showed the morphology of SCM **3** (Figure 2A). A core–shell phase-separated structure of the SCM domains was observed. The hydrophobic core containing Rh-TsDPEN appeared darker in the cryo-TEM images.

**Figure 3:**
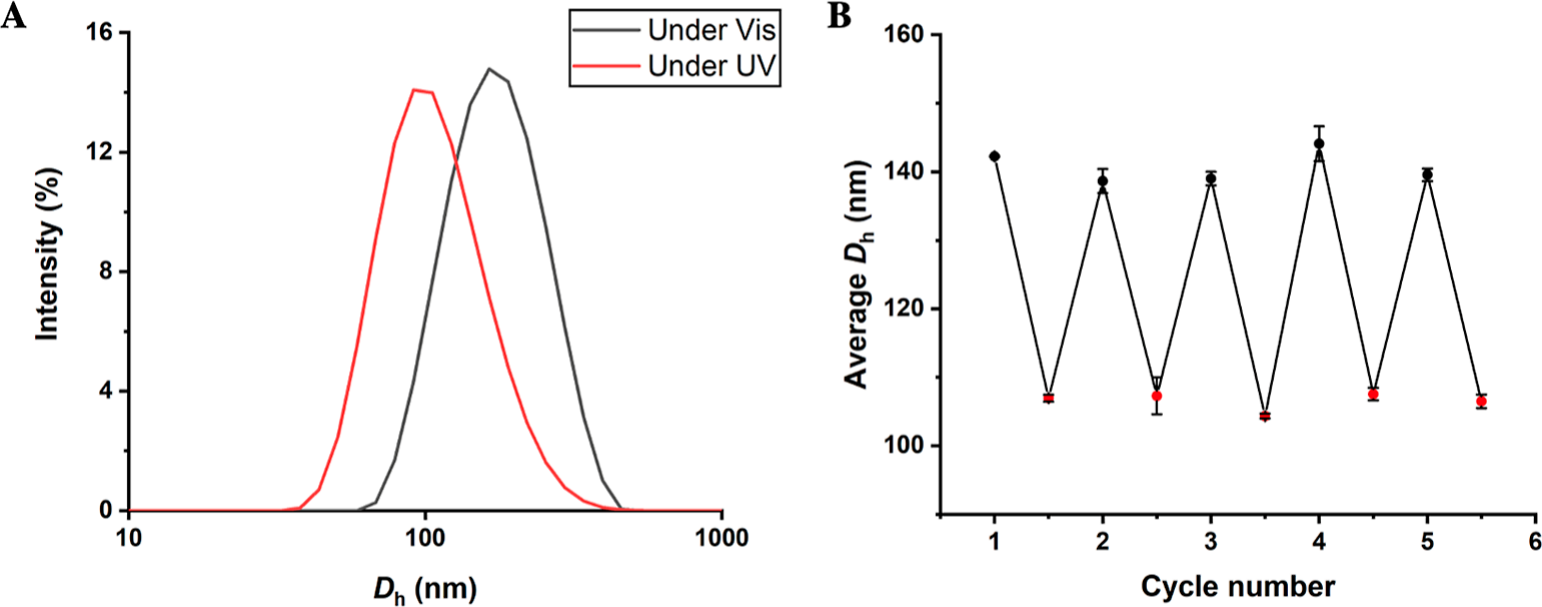
Micelle size investigation of SCM **3**. (A) DLS traces of SCM **3** under UV light irradiation (PDI of 0.203) and under visible light irradiation (PDI of 0.177). (B) *D*_h_ of five consecutive light irradiation cycles determined by DLS. Black dots represent *D*_h_ under visible light and red dots are *D*_h_ under UV light exposure.

DLS of SCM **3** confirmed that the reversible photoswitchable behavior was retained after catalyst immobilizations (Figure 3). The same UV/vis cycle as described for SCM **2** was performed on the micelle solution of SCM **3** at 1.0 mg/mL in water and evaluated using DLS. The *D*_h_ of SCM **3** under visible light was measured to be between 138 and 147 nm. Upon irradiation with UV light, DLS measurements showed an *D*_h_ of 106 ± 4 nm. The increase in the micelle size of SCM **3** compared with SCM **2** is due to catalyst immobilization, resulting in a more hydrophobic micellar structure, resulting in larger *D*_h_ on the DLS.^41^

SCM **3** retained the LCST behavior observed with SCM **2** as determined through turbidimetry measurements by UV/vis spectroscopy, DLS (Figure 1) and cryo-TEM images (Figure 2A). DLS allows for a measurement of the nanostructure size over time. SCM **3** was dispersed in water at 1.0 mg/mL and filtered with a 0.45 μm syringe filter for DLS tests. The micelle *D*_h_ remained stable when heating at 40 °C, below the LCST. The micelle size, as measured by DLS, was 129 ± 4 nm (PDI = 0.314) after heating at 40 °C for 5 h, 130 ± 2 nm (PDI = 0.240) after heating for 24 h (Figure S11) and remained unchanged at 130 ± 2 nm (PDI = 0.298) after heating for 48 h. When temperature above the LCST, the micelle *D*_h_ decreased with heating time at 60 °C and stabilized at 78 ± 5 nm after heating for 15 h (Figure 1B,C). The PDI at each time point is below 0.200, as shown in Table S1. The *D*_h_ of SCM **3** decreased by 50 nm from 143 ± 2 nm when heating at a temperature at 60 °C above the *T*_cp_. To further confirm the cyclic switching behavior and stability as a nanoreactor, we tested the *D*_h_ of SCM **3** under consecutive heat–cooling cycles (Figure 1D). The micelle solution was stirred at room temperature for 24 h and then heated at 60 °C for another 24 h to obtain a stable micelle solution. Subsequently, the micelle solution was cooled to room temperature for a second cycle. The *D*_h_ of SCM **3** was 140 ± 4 nm at room temperature and decreased to 78 ± 5 nm at 60 °C. The change in micelle *D*_h_ was consistent for three heat–cooling cycles, indicating the reversible thermoresponsive nature of SCM **3**. SCM **3** was then dispersed in water at 1.0 mg/mL for cryo-TEM. The cryo-TEM images further confirmed the micellar structure of SCM **3** at 60 °C and the change in micelle morphology (Figures 2A, S12, and S13). Statistical size distribution is based on a sample size of at least 120 micelles in cryo-TEM (Figure 2B). The *z*-average diameter for the micelles, calculated from the distribution, was 138 nm at room temperature and 70 nm at 60 °C. These results demonstrate that temperatures above *T*_cp_ caused shrinkage of the micelles. There was no aggregation observed at 60 °C, confirmed by both DLS and cryo-TEM images, indicating a typical transition of the polymer chains from hydrophilic corona to hydrophobic globule.^69^

**Figure 4:**
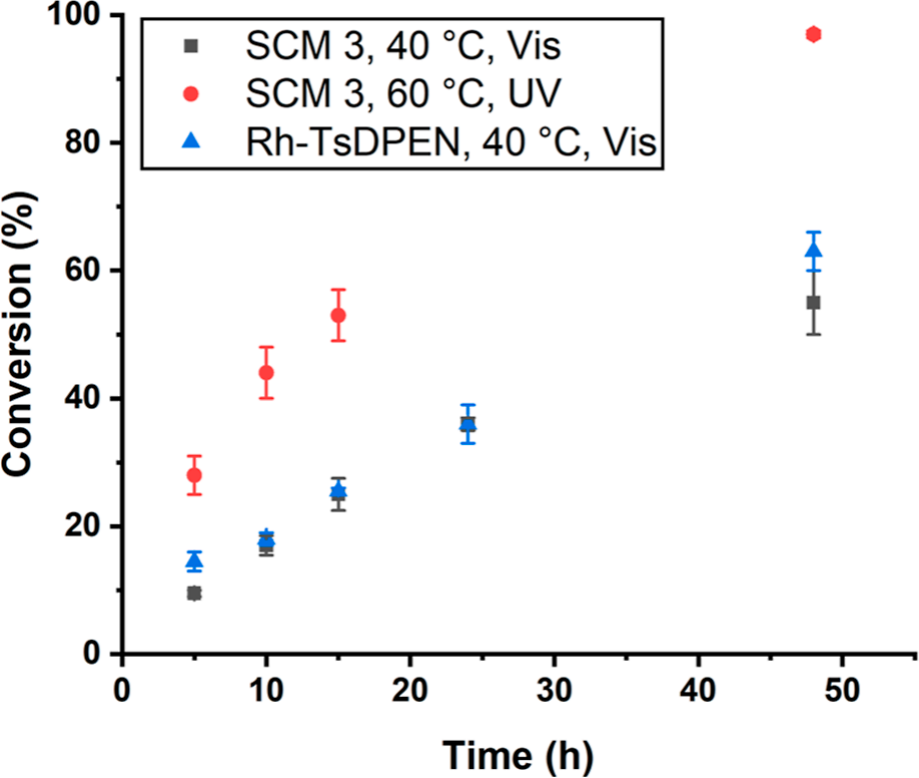
Conversion vs time for Table 1, entries 1, 3, and 5. SCM **3** and the Rh-TsDPEN-catalyzed ATH of acetophenone. All reactions were performed on a 4 μmol substrate scale in 0.6 mL of water, Rh loading was 5% with 10 equiv of HCOONa.

To compare the ATH using supported SCM **3** as catalyst, we first explored the catalytic activity of unsupported Rh-TsDPEN as catalyst for the ATH at temperatures below and above the *T*_cp_ of SCM **3**, 40 and at 60 °C, respectively. With a catalyst loading of 5 mol %, unsupported Rh-TsDPEN catalyzed the ATH of acetophenone at 40 °C with 63% conversion over 48 h. When the temperature was increased to 60 °C, the conversion after 48 h increased by 5 percentage points to 68% (Table 1, entries 1 and 2). By confirming the thermoresponsiveness and photoresponsiveness of SCM **3**, we hypothesized that SCM **3** regulates the channeling of substrates and reagents in ATH through the utilization of temperature and light triggers. To confirm our hypothesis, we investigated the catalytic activity of SCM **3** in the ATH using the same reaction condition. With the same catalyst loading, the ATH conversion using SCM **3** increased by 15 percentage points when the temperature was changed from 40 to 60 °C, a larger increase than the unsupported catalyst (Table 1, entries 3 and 4). To further validate our hypothesis, we investigated the ATH conversion of 1-tetralone using unsupported Rh-TsDPEN as a catalyst. The conversion was 35% at 40 °C and 41% at 60 °C. By using SCM **3** as catalyst, the conversion increased by 27 percentage points. The increase was higher compared to using the unsupported catalyst due to the presence of the thermoresponsive outer layer. Increasing the reaction temperature to 60 °C above the *T*_cp_ leads to a hydrophilic-to-hydrophobic transition of the SCM corona. According to the previous DLS measurements, a shrinkage in SCM size is hypothesized to shorten the substrate diffusion distance from the hydrophilic corona to the hydrophobic core, facilitating faster transit to the catalytic site. Consequently, a higher reaction conversion is reached in 2 days. An alternative explanation could be that elevating the reaction temperature results in an increase of the hydrophobicity of SCM **3**, thereby amplifying the concentration of involved substrates, resulting in an increase of the overall flux and a higher conversion. This ATH with responsive SCM **3** as nanoreactor can be suppressed or accelerated depending on two types of stimuli, temperature, and light. We hypothesize that the lower conversion at 40 °C under visible light (Table 1, entries 3 and Figure 4) can be attributed to the hydrophobic spiropyran cross-linking layer preventing the diffusion of the hydrogen donor, HCOONa, into the core (Scheme 1C). Under UV light at 60 °C, however, catalysis was accelerated with 97% yields and excellent enantioselectivities (99% ee) (Table 1, entries 5 and Figure 4). The conversion increased by 27 percentage points compared to that of the reaction under visible light (Table 1, entry 4). Under UV light irradiation, the spiropyran cross-linking layer isomerizes to merocyanine, resulting in an increase in hydrophilicity and ultimately an increase in permeability for HCOONa (Scheme 1C). The kinetic data (Figure 4) and the catalytic tests of the ATH (Table 1) demonstrate that the hydrophobicity change occurring in the corona and shell layer of SCM **3** results in a difference in substrate conversion. This supports our hypothesis that SCM **3** regulates the channeling of substrates and reagents using temperature and light triggers.

**Table 1:**
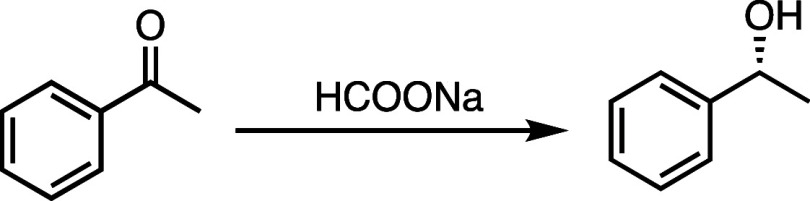
ATH Catalytic Tests of Acetophenone^a^

entry	catalyst	temperature (°C)	light	*D*_h_ (nm)^b^	conversion (%)^c^	ee (%)^d^
1	Rh-TsDPEN	40	visible		63 ± 3	97
2	Rh-TsDPEN	60	visible		68 ± 1	99
3	SCM **3**	40	visible	128 ± 4	55 ± 5	98
4	SCM **3**	60	visible	78 ± 5	70 ± 3	98
5	SCM **3**	60	UV	58 ± 3	97 ± 3	99
6^e^	SCM **3**	60	UV		95 ± 2	98

a Reaction condition: reactions were performed on a 4 μmol substrate scale in 0.6 mL of water. Rh loading was 5% with 10 equiv of HCOONa in open air for 2 days.

b Micelle solution was heated and under visible/UV light irradiation overnight, *D*_h_ was determined by DLS.

c Conversions were determined by ^1^H NMR spectroscopy.

d ee was determined by chiral HPLC analyses.

e Conversion was determined using nanoreactors that had been recycled three times.

We investigated the recovery and recycling of SCM **3** using the ATH of acetophenone. Because of the cross-linking layer stabilizing the SCM structure, the SCM-supported catalysts can be easily recovered 3 times using dialysis without a significant loss of reactivity (Table 1, entry 6).

Due to the thermoresponsive and photoresponsive hydrophobicity transition in separate layers of SCM **3**, we hypothesized that SCM **3** might display substrate-selective behavior based on hydrophobicity regulated by temperature and light wavelength. Here, substrate selectivity refers to the ability of the catalyst to preferentially convert one substrate over another based on their relative hydrophobicity. When temperature rises above the polymer’s LCST, the thermoresponsive hydrophilic corona becomes more hydrophobic, which should accelerate the channeling of more hydrophobic substrates to the catalytic core, resulting in higher conversions in the ATH (Scheme 1A). In contrast, under UV light irradiation, the SCM cross-linking layer isomerizes from spiropyran to merocyanine, increasing the hydrophilicity. The cross-linked layer induces differences in the partitioning of substrates, allowing less hydrophobic substrates to channel the catalytic core faster, leading to higher conversions in the ATH (Scheme 1C). Upon UV light irradiation, conversion promotes less hydrophobic substrates. Temperature elevation, however, has the opposite effect, which promotes conversion of more hydrophobic substrates. Based on the results described above for acetophenone, optimal condition for SCM **3** catalyzing ATH is under UV light irradiation at 60 °C. Therefore, we conducted a substrate screen focusing on “conversion difference” rather than “conversion” under various conditions to elucidate the selectivity of the SCMs.

The catalytic activity and selectivity of SCM **3** were examined for the ATH of a series of aromatic ketones, from less hydrophobic to more hydrophobic (Tables 2 and 3). With a catalyst loading of 5 mol %, SCM **3** showed substrate selectivity based on temperature and light wavelength. We used visible light for all substrates and heated the reaction mixtures at 40 and 60 °C (Table 2). After 48 h, the reaction conversion of 4-acetylbenzoic acid (Table 2, entry 1) increased by 3 percentage points with a temperature increase from 40 to 60 °C. Similarly, 1-(pyridin-3-yl)ethenone (Table 2, entry 2) conversion increased by 2 percentage points; the hydrophilicity of both substrates and the small conversion increase demonstrate the barrier of the hydrophobic outer layer toward these less hydrophobic substrates at 60 °C. Decreasing the polarity along the benzene side-chain of the substrate ketones resulted in an increase in conversion. The conversion for acetophenone increased by 15 percentage points (Table 2, entry 6). For larger ring systems such as 2-acetylanthracene and 1,3-diphenyl-1-butanone (Table 2, entries 9 and 10), the conversions at 40 and 60 °C were both lower than for the smaller substrates acetophenone. We suggest that the reason is size and steric hindrance,^24^ but the total conversion increased by the largest percentage, 42 percentage points and 49 percentage points, respectively—likely due to the hydrophobicity of both substrates. Upon comparison of entries 9 and 10 in Table 2, while having similar substrate molecular sizes (as demonstrated by their respective 3D models in Figure S36), 1,3-diphenyl-1-butanone exhibits higher hydrophobicity. This difference in hydrophobicity correlates with an increase in conversion with temperature for 1,3-diphenyl-1-butanone (49%) compared to 2-acetylanthracene (42%). These results indicate that substrate hydrophobicity has a significant effect on the observed differences in conversion than substrate size. It is noteworthy that elevating the temperature to 60 °C does not change the spiropyran of the cross-linked shell layer. The material still shows a peak at 550 nm in the UV/vis spectra (Figure S14). This demonstrates that changes in temperature do not alter the diffusion ability of the photoresponsive shell of SCM **3**. ATH using SCM **3** as a nanoreactor exhibits a substrate selectivity based on hydrophobicity induced by temperature: at temperatures above the *T*_cp_, the outer layer of the SCM becomes more hydrophobic, leading to higher conversions for more hydrophobic substrates.

**Table 2:**
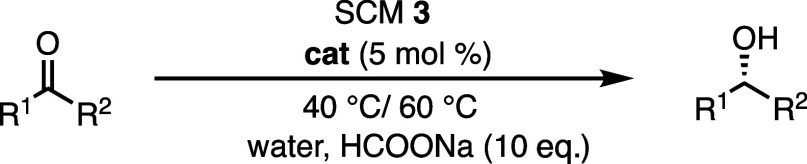

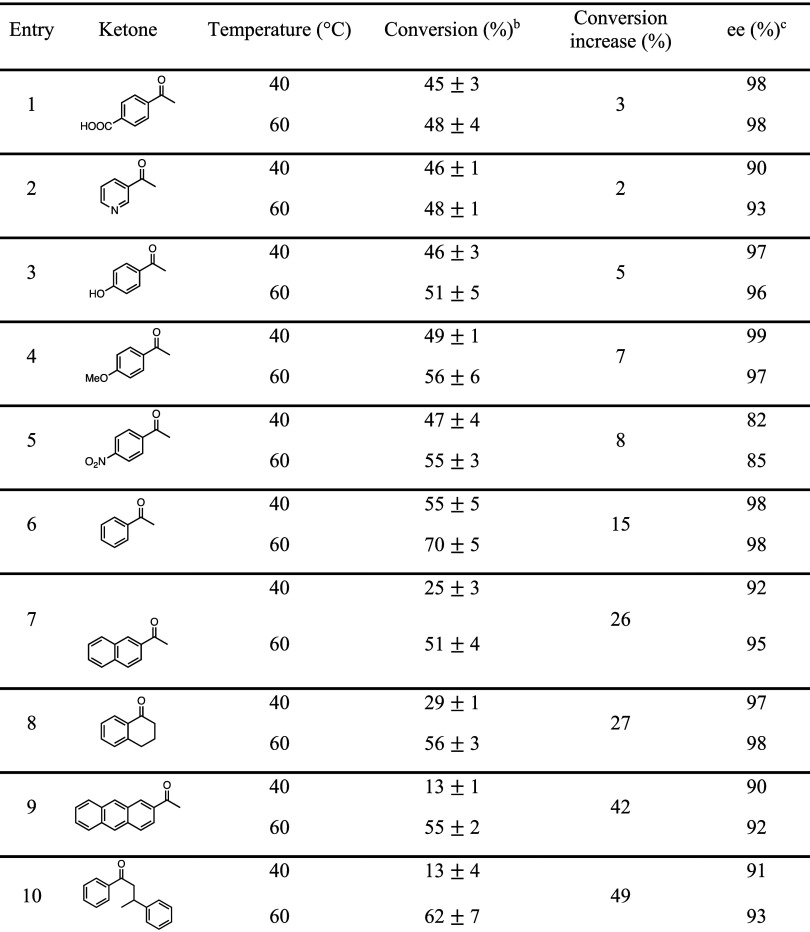
SCM **3** Catalyzed ATH of Ketones in Water at 40 °C/60 °C under Visible Light^a^

a Reaction conditions: reactions were performed on a 4 μmol substrate scale in 0.6 mL of water, Rh loading was 5% with 10 equiv of HCOONa in open air at 60 °C for 2 days.

b Conversions were determined by ^1^H NMR spectroscopy. Three sets of parallel experiments were conducted, and the conversion results were averaged.

c ee was determined by chiral HPLC analyses.

**Table 3:**
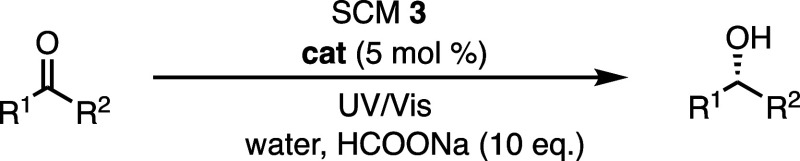

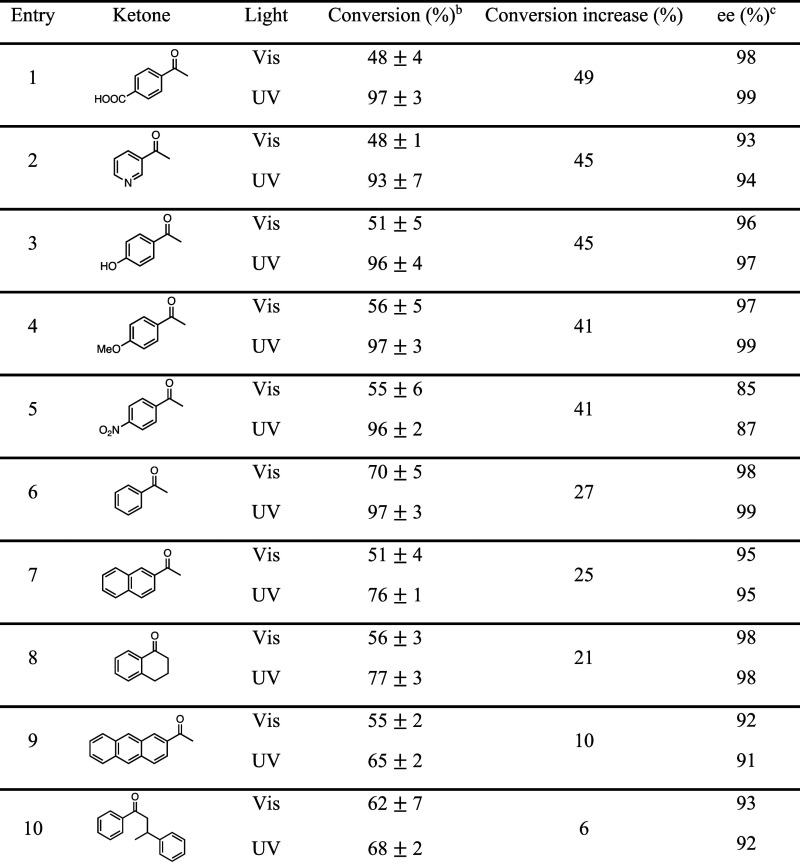
SCM **3**-Catalyzed ATH of Ketones in Water under UV/Visible Light at 60 °C^a^

a Reaction conditions: reactions were performed on a 4 μmol substrate scale in 0.6 mL of water, Rh loading was 5% with 10 equiv of HCOONa in open air at 60 °C for 2 days.

b Conversions were determined by ^1^H NMR spectroscopy. Three sets of parallel experiments were conducted, and the conversion results were averaged.

c ee was determined by chiral HPLC analyses.

To further examine our hypothesis of phototriggered substrate-selective ATH, the same library of ketones was transformed at a fixed temperature of 60 °C but under changing the wavelength of light irradiation. Using SCM **3** as nanoreactor with catalyst loadings of 5 mol %, light wavelength was altered from 550 nm (visible light) to 350 nm (UV light) (Table 3). There is an increase in conversion for all substrates when switching from visible light to UV light because the phototriggered transition from spiropyran to merocyanine enables the diffusion of HCOONa into the core. The availability of HCOONa in the core remained the same for all substrates.

Next, we compared the conversion increases among the substrates. We first explored the ATH of less hydrophobic aromatic ketones, with polar side groups (Table 3, entries 1–5), using SCM **3**. Under UV light irradiation, the ketones were almost fully transformed to the corresponding secondary alcohols with quantitative conversions. Among them, the conversion of 4-acetylbenzoic acid increased the most while switching from visible light to UV light (Table 3, entry 1), from 48 to 97%, a 49% increase in conversion, indicating that SCM **3** favored less hydrophobic substrates under UV light because of the transformed hydrophilic merocyanine cross-linking layer. The conversion of acetophenone under UV light reached 97% (Table 3, entry 6), but the conversion increase was only 27%, which is smaller than for 4-acetylbenzoic acid. We hypothesize that the hydrophobic spiropyran cross-linking layer under visible light prevents the less hydrophobic substrate 4-acetylbenzoic acid from entering the catalytic core, resulting in lower initial conversions and a larger conversion increase upon UV light irradiation. Increasing the number of carbons in the target ketone resulted in a smaller increase on conversions under UV light irradiation (entries 6–8), indicating that the polar merocyanine in the shell hinders the more hydrophobic substrates. For the most hydrophobic substrate, 1,3-diphenyl-1-butanone, the increase in conversion was only 6% (Table 3, entry 10). These results support the conclusion that SCM **3** performs phototriggered substrate-selective ATH, allowing higher conversion of less hydrophobic substrates under UV light. For less hydrophobic substrates, elevated temperature suppresses substrate channeling, while UV irradiation accelerates substrates entering the core and yields higher conversions in comparison with visible light irradiation. Conversely, for the more hydrophobic substrates, an increase in temperature results in higher conversions, while UV light irradiation suppresses the conversions.

## Conclusions

In summary, we fabricated thermo- and photoresponsive SCMs as nanoreactors for the substrate-selective ATH in water. Immobilized chiral rhodium catalysts at the core of the smart nanoreactors catalyze ATH for a series of aromatic ketones. We equipped the SCM-based nanoreactors with a dual switch: one light activated in the cross-linking shell, and the other heat activated in the outer corona. The two switches allow for tunable reaction pathway control by changing the hydrophobic nature in the corona and shell of the SCMs. Light and temperature, as triggers, dynamically modulate the substrate selectivity of the ATH, preferentially converting one substrate over another based on their hydrophobicity. At higher temperature (60 °C), the thermoresponsive outer layer of the SCMs channels more hydrophobic substrates, resulting in higher conversions. Under UV light, less hydrophobic substrates can channel through the hydrophilic merocyanine cross-linking layer into the catalytic core, exhibiting a higher conversion. The smart SCM nanoreactors mimic the substrate channeling of cells in nature which coordinate metabolic pathways *via* multiple external stimuli. We suggest that our strategy may be extended to smart drug carriers for different controlled release applications.^70^ Future research in our group will focus on preparing multiple stimuli-responsive compartmentalized nanoreactors to regulate incompatible tandem reactions.

## Supporting Information Available

Synthetic procedures and analytical data (PDF)

## Abbreviations

NMR: nuclear magnetic resonance
FTIR: Fourier-transform infrared spectroscopy
ee: enantiomeric excess
UV: ultraviolet light
