# Supplementary material for: Substrate Channeling in Compartmentalized Nanoreactors
Source: Macromolecules. 2024 Jul 3;57(14):6805–15. doi: 10.1021/acs.macromol.4c00697 (PMC11270995; doi:10.1021/acs.macromol.4c00697)
Supplement: Supplementary file 1 — ma4c00697_si_001.pdf [file ma4c00697_si_001.pdf]

Supporting Information

Substrate Channeling in Compartmentalized Nanoreactors

Fangbei Liu, Peiyuan Qu, Jeremy Weiss, Kunhao Guo and Marcus Weck*

Molecular Design Institute and Department of Chemistry, New York University
New York, NY 10003, USA
marcus.weck@nyu.edu

Contents

1. Materials and Methods 2

2. Synthetic Procedures 4

2.1 Polymerization & Post-polymerization Modifications 4

2.1.1 CROP of triblock copolymer 1 4

2.1.2 Self-assembly protocol 4

2.1.3 Synthesis of SCM 2 through CuAAC 7

2.1.4. Synthesis of SCM 3 through thiol-ene chemistry 10

3. General Procedures for Micelle-Supported ATH Catalysis 14

3.1 General procedures and kinetic data for micelle-supported asymmetric transfer hydrogenation (ATH) 14

3.2 Analytic data of ATH products 15

4. References 35

1. Materials and Methods

2-Tridecanyl-2-oxazoline (TridecanylOx)[1], 2-(But-3-yn-1yl)-4,5-dihydrooxazole (ButynOx)[2], alkene functionalized Rh-TsDPEN catalyst[3], and bi-functionalized spiropyran cross-linker **N_3-SP-N_3**[4] were synthesized based on adapted literature procedures. All other reagents were purchased from Sigma Aldrich, Alfa Aesar, TCI America, Enamine, or Frontier Scientific and used as received unless otherwise stated. 2-isopropyl-2-oxazoline (iPrOx) was purchased and distilled over CaH_2 and stored under argon before polymerization. Acetonitrile and chlorobenzene were distilled over CaH_2 and stored under argon and molecular sieves (4 Å). Methyltriflate was distilled over CaH_2 and stored under argon at 4 °C. Flash column chromatography was performed using silica gel 60 Å (230-400 mesh) from Sorbent Technologies.

NMR spectroscopic characterization was conducted on a Bruker Avance 400, 500, 600 or 800 MHz spectrometer. Chemical shifts are reported in ppm and referenced to solvent residual peaks. Splitting patterns are reported as singlet (s), broad singlet (br s), doublet (d), broad doublet (br d), doublet of doublets (dd), triplet (t), broad triplet (br t), quartet (q) and unresolved multiplet (m).

Mass spectra of samples in methanol were acquired with an Agilent 6224 Accurate-Mass TOF/LC/MS Spectrometer.

Gel-permeation chromatography (GPC) was carried out using a Shimadzu pump coupled to a Shimadzu RI detector controlled by an EZStart program. A set of polymer standards columns (AM GPC gel, 10 μm, precolumn, 500 Å and linear mixed bed) was used with a 0.03 M LiCl solution in *N,N*-dimethylformamide at a flow rate of 1 mL/min at 60 °C. The system was calibrated with poly(styrene) standards (EasiCal, Agilent Technologies, Santa Clara, CA). The injection volume was 100 μl and the flow rate was 1 mL/min. M_n^{app} and dispersity (*Đ*) represent the apparent number-average molecular weight and dispersity index respectively.

Hydrodynamic diameters of cross-linked and uncross-linked micelles were determined at 25 °C by dynamic light scattering (DLS) using Malvern Zetasizer nano series with a 663 nm module.

Analytical high performance liquid chromatography (HPLC) was performed on an Agilent 1200 series with a diode array detector (samples analyzed at 210 nm and 280 nm). A Chiracel OD column (Chiral Technologies, Inc.) was used for ee determination.

UV-Vis spectra were recorded with a Cary 100 Bio UV-VIS Spectrophotometer (No. EL06023666) coupled to a Cary Temperature Controller (No. EL06023011). The spectra were recorded without stirring at a wavelength of 700 nm from 20-100 °C, at a heating rate of 1 °C min^{-1}, with a sample concentration of 0.1 mg/mL in a 1400 μL micro cuvette with stopper (Thor labs #CV10Q1400S)

Dialysis was performed using a Spectra/Por 6 dialysis membrane with a MWCO of 2000 Da or 1000 Da.

A 365 nm UV lamp (15W UVP Black Ray UV Bench LampXX-15L) was used for the thiol-ene reactions.

A Pen-Ray light source P/N 90-0019-01 (wavelength = 365 nm, power = 50/60 Hz, intensity = 145 μW/ cm^2) was used for all UV experiments.
Inductively coupled plasma mass spectrometry (ICP-MS) was carried out by Robertson Microlit Laboratories, Inc.

Cryo-TEM grids (quantifoil R1.2/1.3 with 300 mesh copper (Cat. #Q350-CR1.3)) were plasma treated for 30 seconds using Denton Vacuum Bench Top Turbo before use. Cryo-TEM grids were prepared in a Leica EM GP at 30 °C with the relative humidity set to 70%. 5 μl of sample was pipetted onto a freshly glow-discharged grid. The sample solution was incubated on the TEM grid for 30 seconds, blotted for 4 seconds before being plunged into liquid ethane that was pre-cooled to -183°C by liquid nitrogen. The cryo-TEM grids were then transferred to and stored in liquid nitrogen. The cryo-TEM grids were transferred in liquid nitrogen into a Gatan 626 cryo-specimen holder and then inserted into the microscope. The specimen temperature was maintained at −170 °C during data collection. Cryo–TEM imaging was performed in a Thermo Fisher Talos 120 C TEM operating at 120 kV on a Gatan One View camera (4k×4k).

FT-IR spectra were recorded on a Nicolet iS50 FT-IR spectrometer, equipped with a Smart iTR ATR accessory.

2. Synthetic Procedures

2.1 Polymerization & Post-polymerization Modifications

2.1.1 CROP of triblock copolymer 1

In glove box, a solution of iPrOx (276 µl, 2.32 mmol) in acetonitrile (1.0 mL) was added in an oven-dried 25 mL Schlenk flask. To initiate the polymerization, methyl triflate (MeOTf) (2.53 µl, 0.0232 mmol) was introduced out of the glove box under argon, and slowly lowered into an oil bath preheated at 140 °C. The reaction was stirred for two hours, and the conversion monitored by ^{1}H-NMR spectroscopy via the disappearance of the monomer signals at 0.8 and 1.7 ppm as well as the appearance of the polymer side-chain signals. After full conversion was confirmed, ButynOx (85.7 mg, 0.696 mmol) was added in glove box and the reaction was stirred for one hour until all monomers was consumed. A solution of TridecanylOx (29.3 mg, 0.116 mmol) in glove box was added to the reaction mixture. The reaction was stirred overnight until full conversion. 20 µl of allyl amine were added to terminate the polymerization and the reaction was stirred for an additional hour at 140 °C. The reaction mixture was dialyzed against methanol and lyophilized from H_2O.

End group analysis by ^{1}H-NMR spectroscopy (Figure S1) showed repeat units for iPrOx (100), ButynOx (30), TridecanylOx (5). ^{13}C-NMR spectroscopy (Figure S2) and IR spectrum (Figure S3) was also obtained. The molecular weight distributions as determined by GPC (M_n^{app} = 8093 Da) was $Đ$ = 1.30 (Figure S4).

2.1.2 Self-assembly protocol

The amphiphilic triblock copolymer with a concentration of 1 mg/mL was stirred overnight and self-assembled into nanostructure in different solvent e.g. water and methanol. The hydrodynamic diameter (D_h) was measured via DLS. The z-average D_h was 65 nm and 5 nm, in water (Figure S5, left) and methanol (Figure S5, right), respectively.

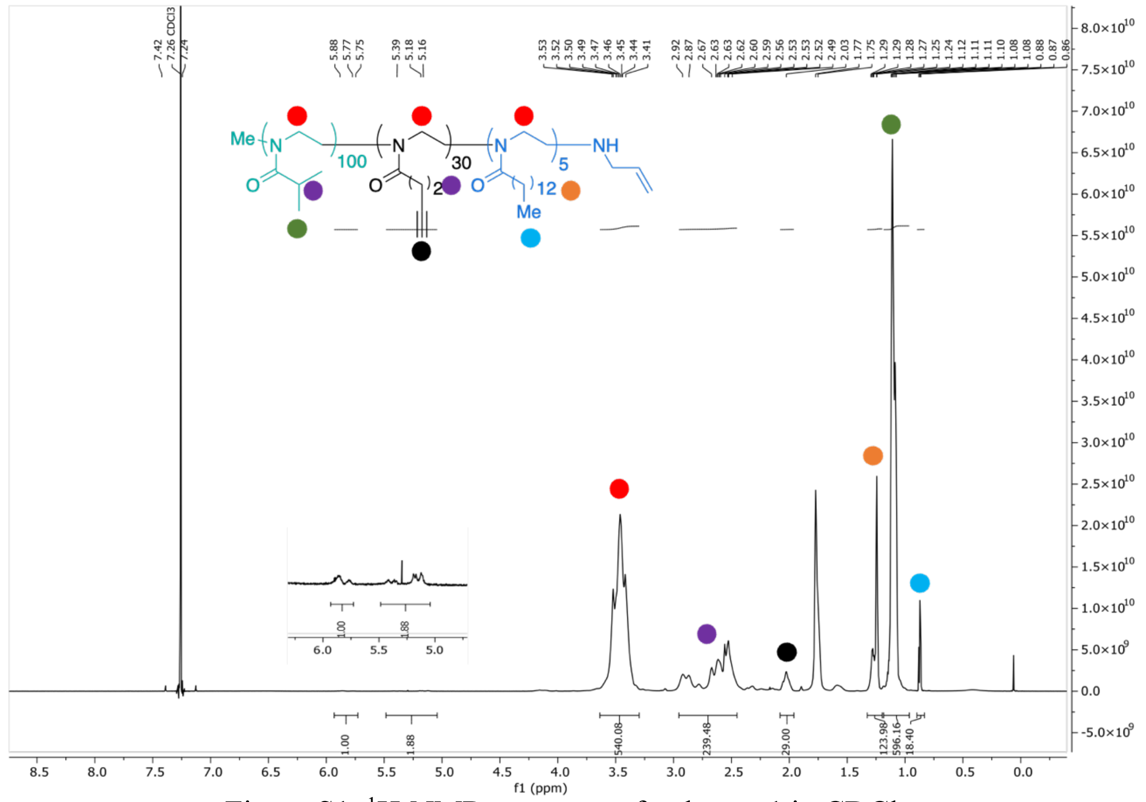

Figure S1. ^{1}H-NMR spectrum of polymer **1** in $CDCl_3$.

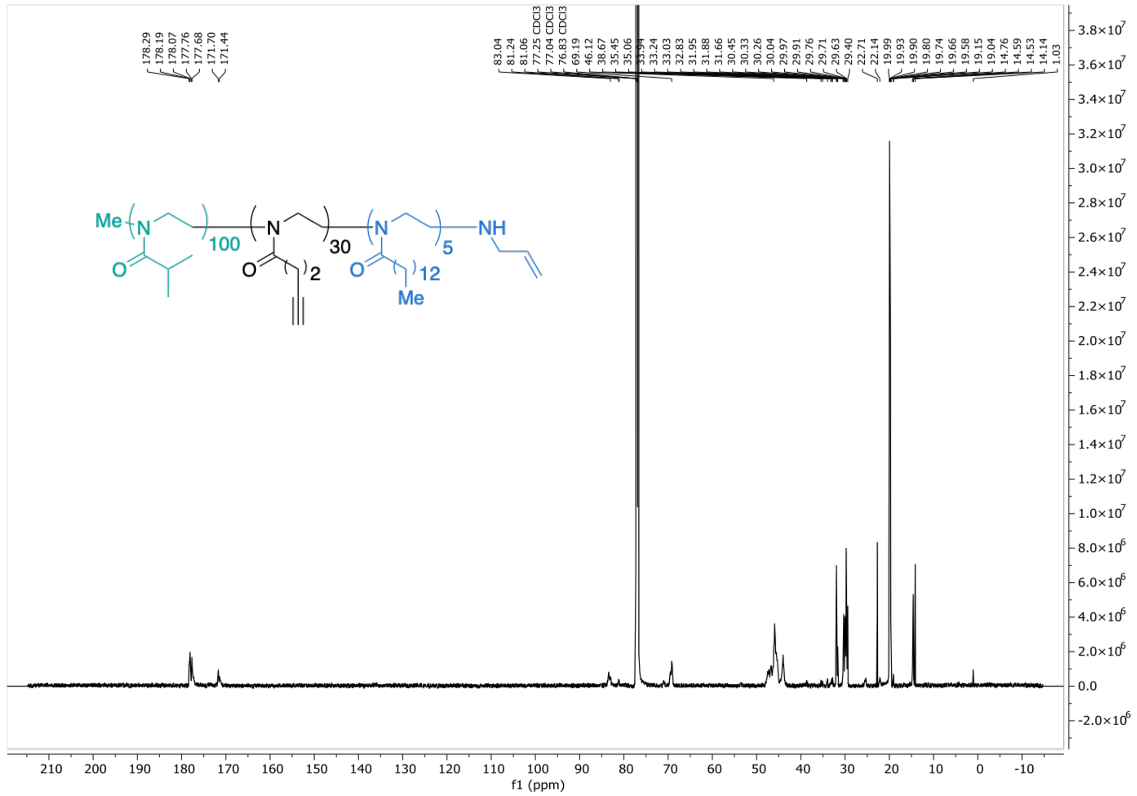

Figure S2. ^{13}C-NMR spectrum of polymer **1** in $CDCl_3$.

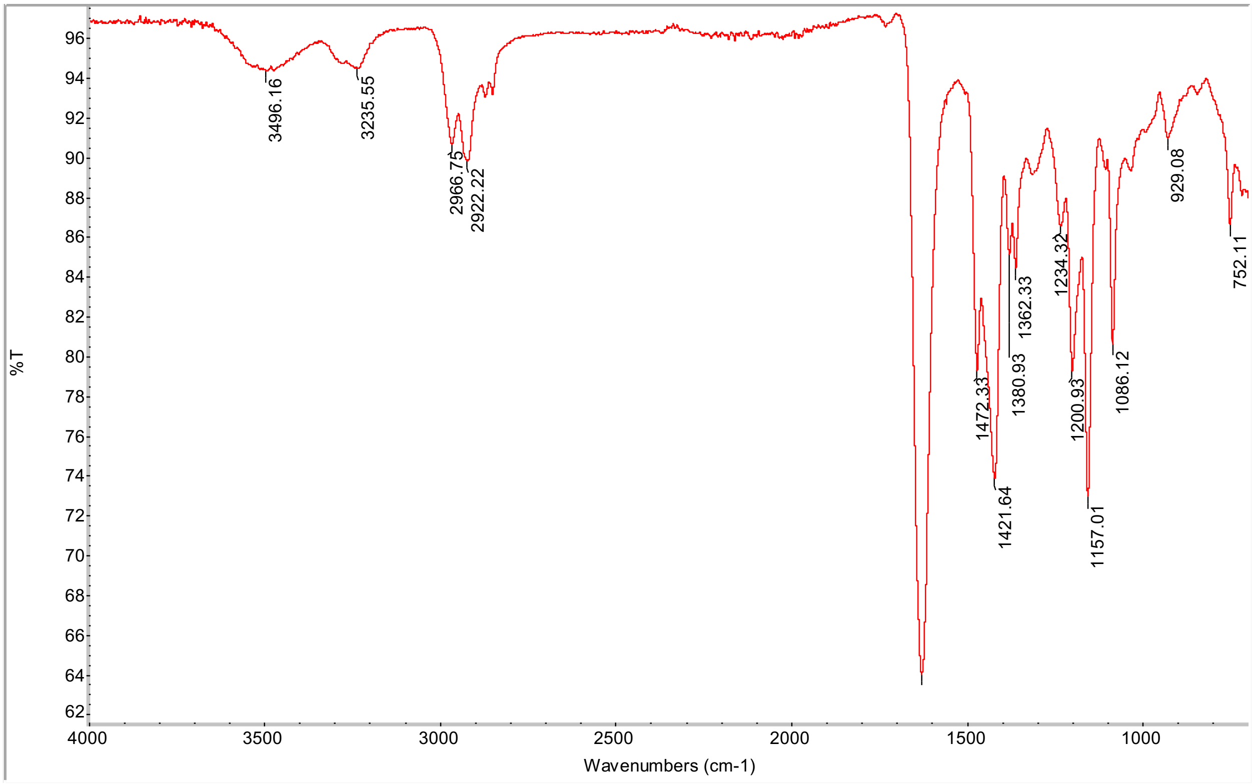

Figure S3. IR spectrum of polymer **1**.

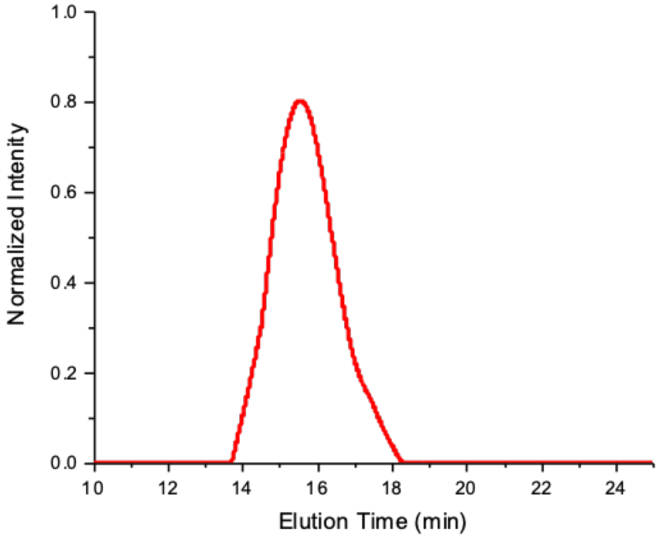

Figure S4. Normalized gel-permeation chromatogram of polymer **1**.

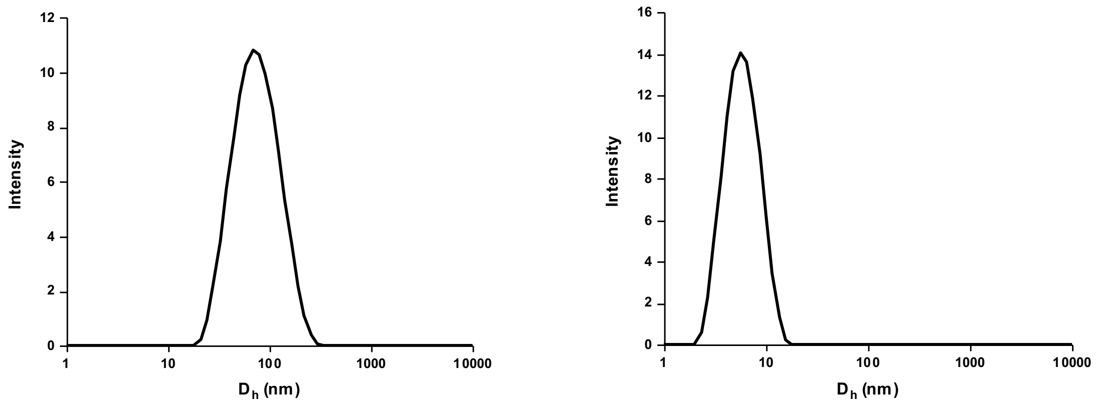

Figure S5. DLS traces of polymer **1** micelle solution in water (left) and methanol (right) at 1.0 mg/mL filtered with a 0.45 μm syringe filter.

2.1.3 Synthesis of SCM 2 through CuAAC

Polymer **1** (100 mg) was self-assembled into a micelle in water overnight with a concentration of 1 mg/mL. It was then transferred to an oven-dried 500 mL Schlenk flask and sodium ascorbate (39.6 mg, 2 equiv. with respect to Cu catalyst), *N, N, N', N'*-tetramethylethylenediamine (TMEDA) (18 μl, 1.2 eq. with respect to Cu catalyst), an acetone solution (5 mL) of cross-linker **N_3-SP-N_3** (43.2 mg, 15 equiv. with respect to polymer) were added. The reaction was degassed via three freeze-pump-thaw cycles, filled with N_2 and an aqueous solution (3.5 mL) of $CuSO_4 \cdot 5H_2O$ (25 mg, 0.5 equiv. with respect to N_3 group) was added into reaction mixture under N_2 protection. The reaction mixture was stirred at room temperature for five days. The complexation and precipitation of Cu by sodium diethyldithiocarbamate was performed to remove all Cu. Excess amount of sodium diethyldithiocarbamate (DDC) (300 mg) was added into the reaction mixture. After stirring for thirty minutes, the reaction mixture was diluted ten-fold. The formed precipitate was carefully filtered off using 0.45 μm syringe filters and the filtrate was concentrated under reduced pressure. The mixture was dialyzed against methanol and DI water and lyophilized from water to afford a yellow power. A high density of covalent crossing-linking of SCM **2** was confirmed by the disappearance of the characteristic alkyne signals at 1.98 ppm in the ^{1}H NMR spectrum, at 84.7 and 71.0 ppm in the ^{13}C NMR spectrum, and the stretch at 3235.6 cm^{-1} in the IR spectrum (Figure S6, S7, S8). The D_h of the nanostructures change (from 5 nm to 63 nm) in methanol also indicated successful covalent cross-linking of the micelles (Figure S10). The photo-responsiveness of SCM **2** was investigated via DLS in a recycling fashion. SCM **2** was dispersed in water at 1 mg/mL for DLS analysis. The micelle solution was exposed to visible light (*l* = 550 nm) for 15 minutes and then switched to UV irradiation (*l* = 350 nm) for 15 minutes. The solution color changes from yellow to purple (Figure S10 inset). Five consecutive UV-Vis switching cycles were performed. The hydrodynamic diameter (D_h) distribution was measured in water via DLS. The micelle solution was filtered with a 0.45 μm syringe filter before DLS measurement. In water, the D_h of SCM **2** under visible light was measured to be between 78 nm and 80 nm. Upon irradiation with UV light, the D_h changed to 68 nm and 70 nm (Figure S9).

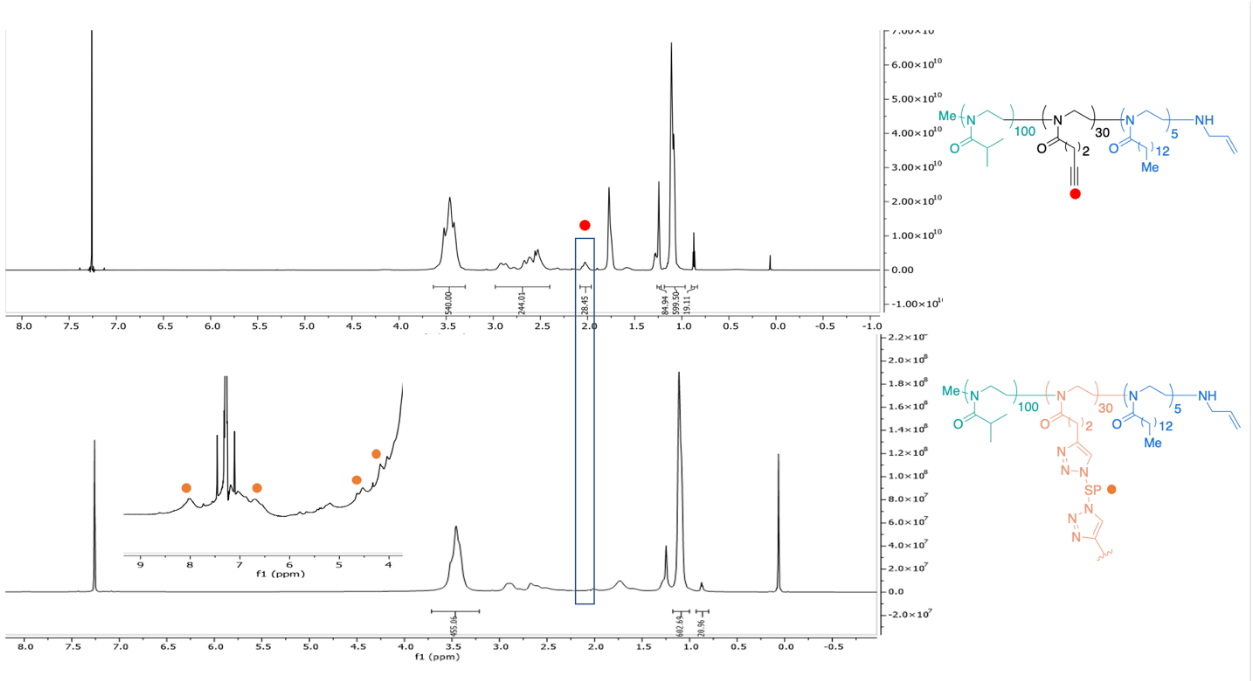

Figure S6. ^{1}H-NMR spectra of SCM **2** in solution of $CDCl_3$ and the comparison of polymer **1**. The alkyne signals at 1.98 ppm disappeared. Inset: enlargement of ^{1}H-NMR spectrum of SCM **2** from 4 ppm to 9 ppm shows the presence of the aromatic SP signals.

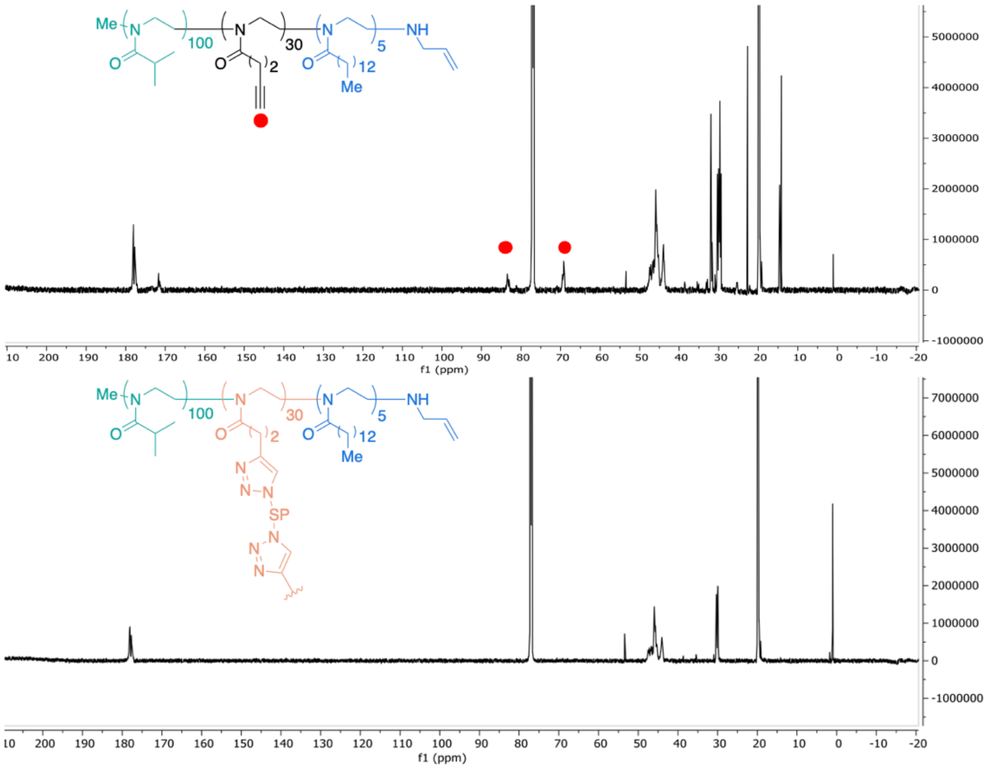

Figure S7. ^{13}C-NMR spectra of SCM **2** in solution of $CDCl_3$ and the comparison of polymer **1**.

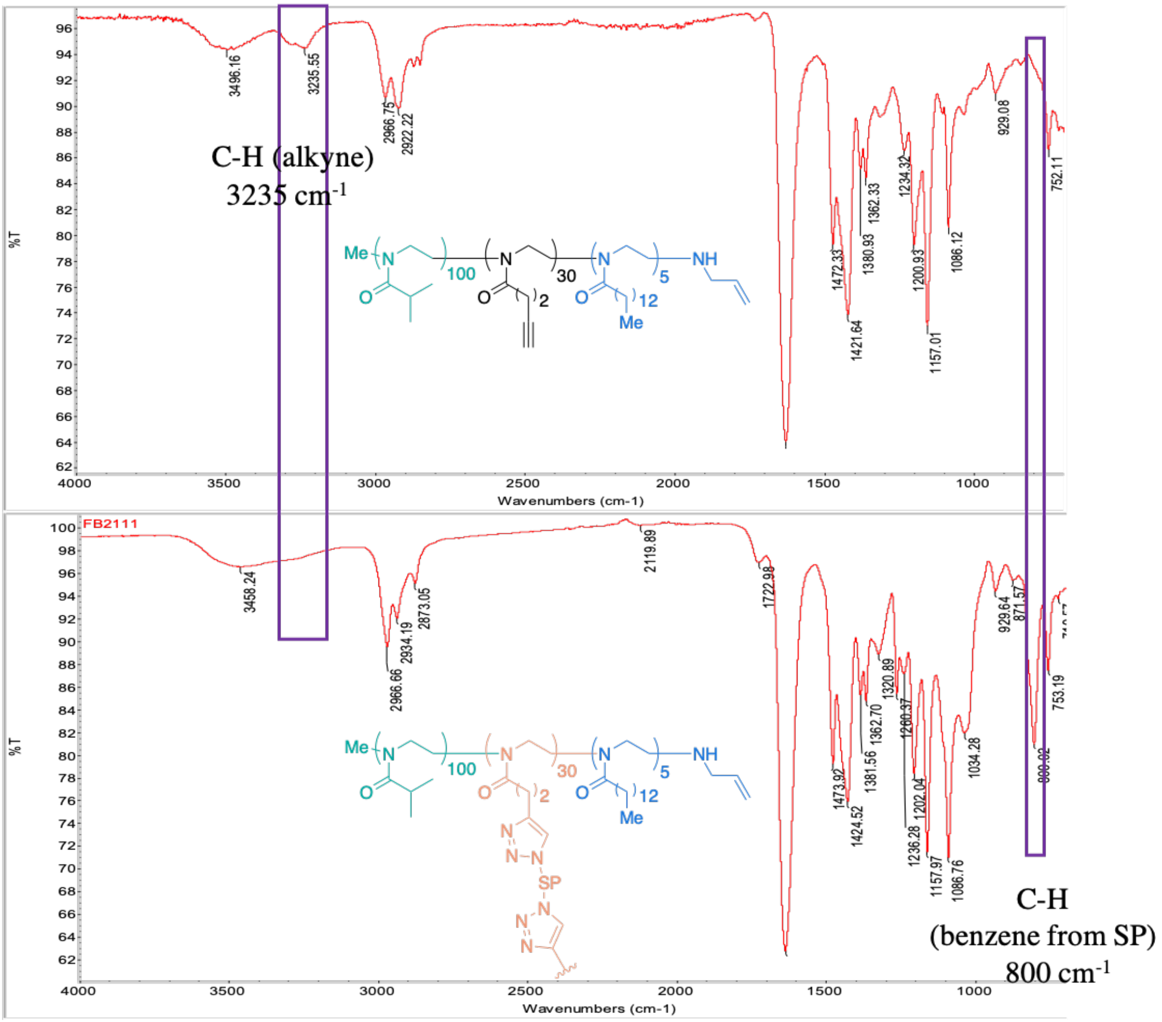

Figure S8. FT-IR spectra of SCM **2** and the comparison with polymer **1**.

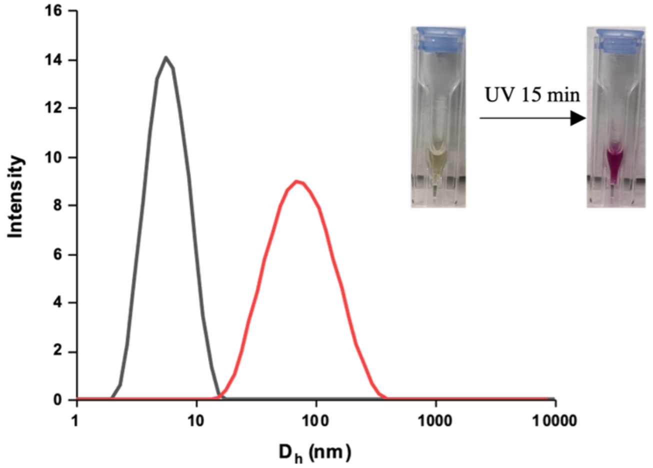

Figure S9. DLS traces of SCM **2** (Red) and comparison with polymer **1** (black) in methanol. Inset: color change of SCM **2** water solution under visible light (yellow) and under UV light exposure for 15 minutes (purple).

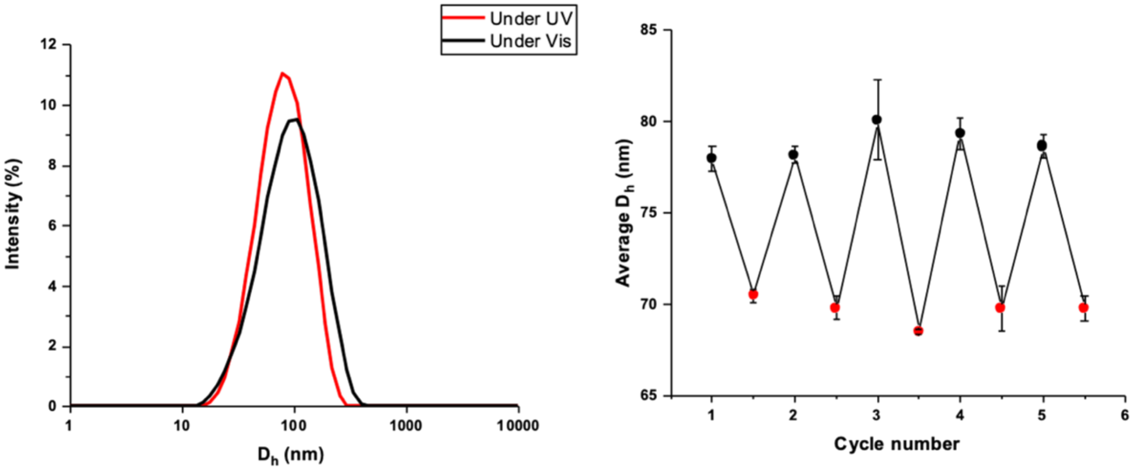

Figure S10. SCM **2** size investigation. Left: DLS traces of SCM **2** water solution under UV light exposure (Red) and under visible light exposure (Black). Right: D_h of five consecutive UV-Vis cycles. Black dots represent D_h under visible light exposure and red dots under UV light.

2.1.4. Synthesis of SCM 3 through thiol-ene chemistry

A solution of SCM **2** (40 mg) in water (40 mL) was prepared and stirred overnight prior to addition to a 100 mL Schlenk flask. Pentaerythritol tetra(3-mercaptopropionate) (4SH) (1 equiv. with respect to alkene groups, 1.22 mg) was added, followed by 2,2-dimethoxy-2-phenylacetophenone (DMPA) (0.2 equiv., 0.128 mg). The reaction was degassed via three freeze-pump-thaw cycles and subjected to 365 nm UV light while stirring for 24 hours at 4 °C. After dialysis against methanol for two days, the methanol was removed via reduced vacuum and the particles were dispersed in 40 mL water and added to a 100 mL Schlenk flask. Olefin functionalized Rh-TsDPEN (3 equiv. with respect to alkene groups) was added, followed by DMPA (0.2 equiv., 0.128 mg). The reaction was degassed via three freeze-pump-thaw cycles and subjected to 365 nm UV light while stirring for 24 hours at 4 °C. After dialysis against methanol and then water for two days, SCMs were dried by lyophilization from water. The Rh content was determined by ICP-MS to be 1.1 %, which corresponds to a degree of functionalization of, on average, 1.70 rhodium complexes per polymer chain. The hydrodynamic diameter (D_h) distribution was measured in water via DLS with a concentration of 1 mg/mL filtered with a 0.45 µm syringe filter. Exposure to UV light lasted for 15 minutes, and the irradiation with Vis was for 15 minutes. In H_2O, the z-average of D_h under visible light exposure fluctuated between 137 nm and 145 nm, while the z-average of the D_h under UV exposure was between 104 nm and 109 nm. The thermoresponsiveness of SCM **3** was confirmed via thermal UV-Vis spectra, DLS and Cryo-TEM. The SCM **3** was dispersed in water at 0.1 mg/mL for thermal UV-Vis and at 1 mg/mL for DLS and Cryo-TEM. The micelle structure of SCM **3** was investigated at room temperature and at 60 °C. The micelle size became stable after heating for 15 hours.

We also explored the UV-Vis spectra of cross-linker **N_3-SP-N_3** under various conditions (Figure S13). When under visible light, the traces are almost same at 40 °C and at 60 °C.

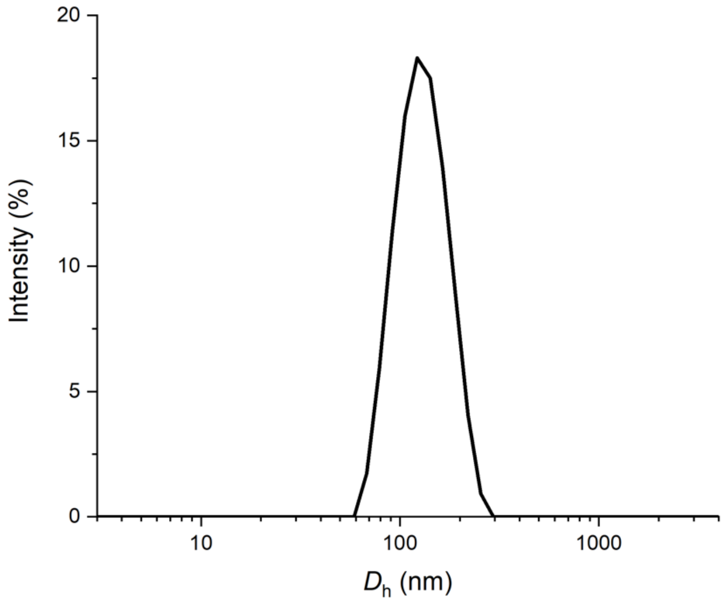

Figure S11. DLS traces of SCM 3 at 40 °C heating for 24 hours (PDI of 0.240).

Table S1. PDI of each point in Figure 1B

Time (h)	5	10	15	24	48
PDI	0.126	0.158	0.084	0.102	0.198

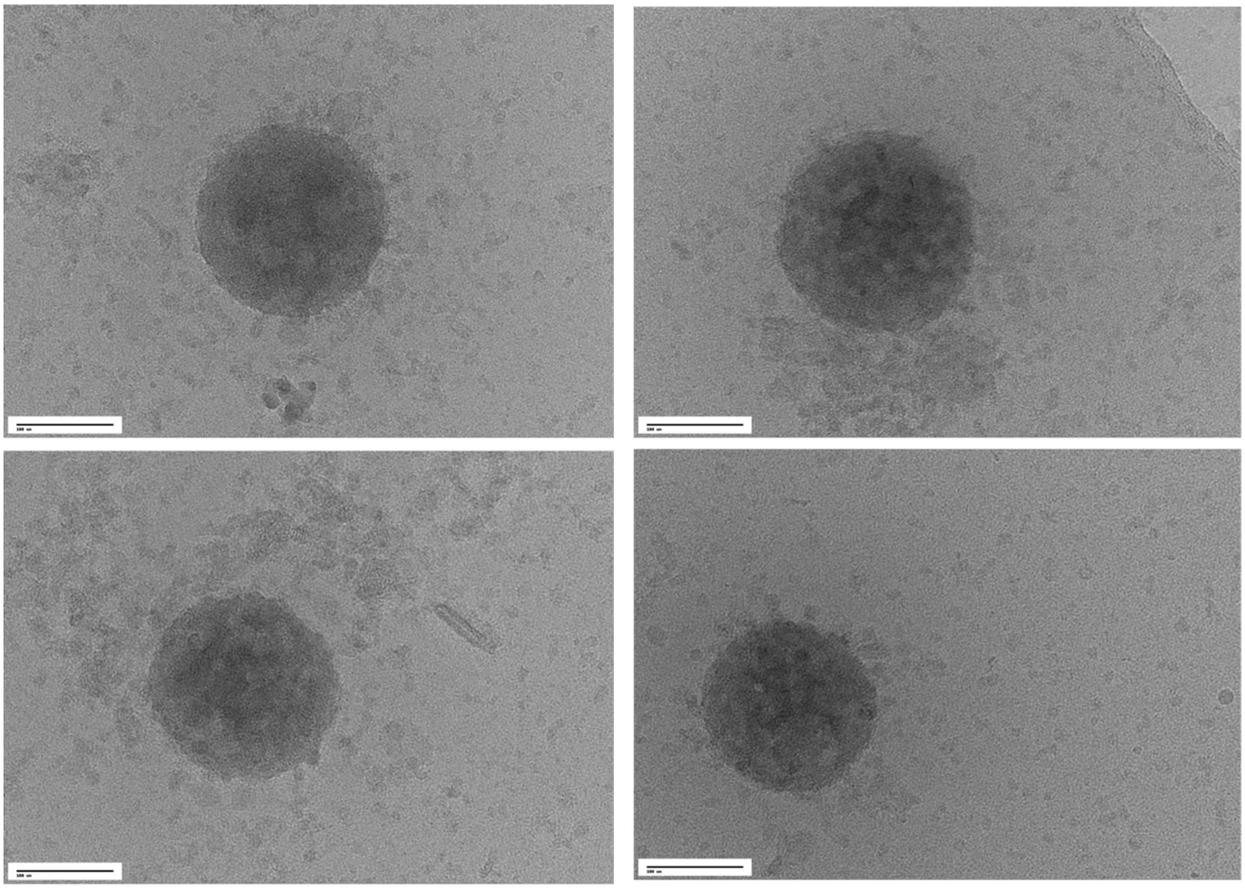

Figure S12. Cryo-TEM images of SCM **3** assemble in aqueous media at room temperature at 1 mg/mL. Scale bars: 100 nm

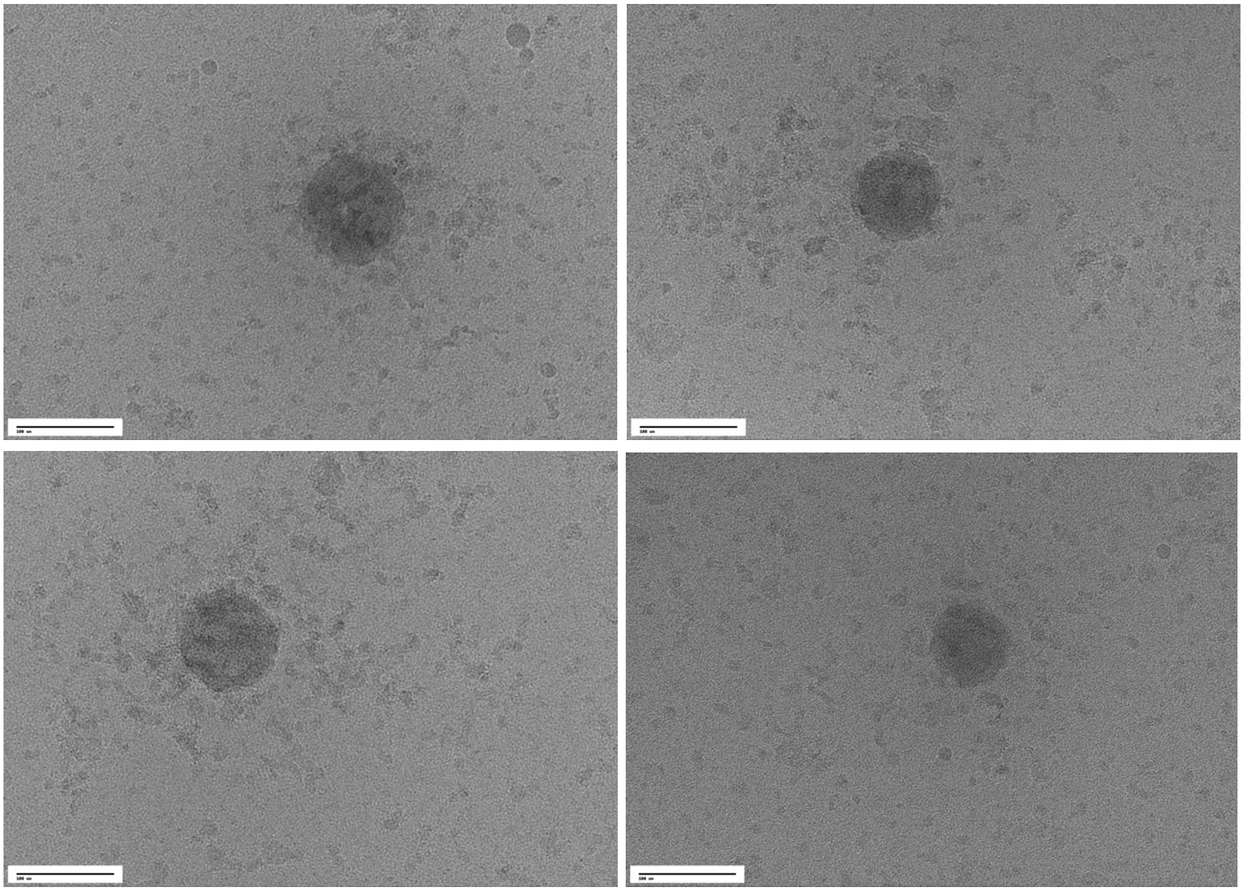

Figure S13. Cryo-TEM images of SCM **3** assemble in aqueous media at 60 °C (solution heated overnight) at 1 mg/mL. Scale bars: 100 nm

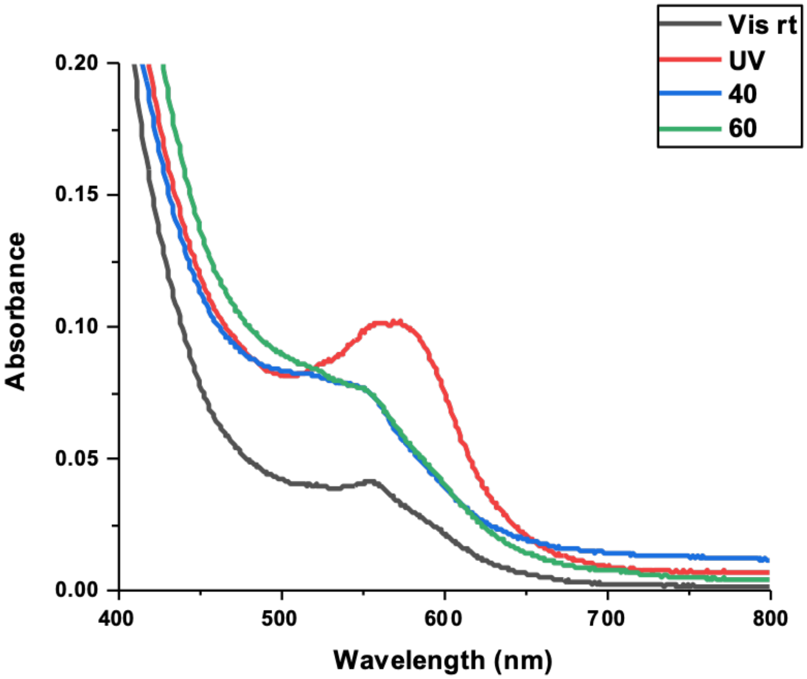

Figure S14. UV-Vis spectra of **N_3-SP-N_3** under various conditions.

3. General Procedures for Micelle-Supported ATH Catalysis

3.1 General procedures and kinetic data for micelle-supported asymmetric transfer hydrogenation (ATH)

SCM **3** (1.9 mg, containing 0.2 μmol Rh-TsDPEN) was weighed into a 4 mL vial. 0.6 mL DI water was added, and the mixture was stirred overnight at room temperature until a uniform micelle suspension was obtained. Acetophenone (acp) (0.5 mg, 4 μmol, 1.0 equiv.) and HCOONa (10 eq. 2.7 mg) were added to the micelle solution. The mixture was stirred at 40 °C or 60 °C under visible light or UV irradiation. Aliquots (0.1 mL) were taken at certain time intervals to monitor the reaction progress. The aliquots were extracted with 0.2 mL EtOAc twice. After removing volatiles, the crude product was dissolved in $CDCl_3$ and filtered through a pipet silica column. With mesitylene (1 equiv.) as an internal standard added, the crude product was subjected to ^{1}H-NMR analysis to determine conversions. Three sets of parallel experiments were conducted, and the conversion results were averaged. After use, the nanoreactors can be recycled via dialysis against methanol and water for two days and then lyophilized.

The conversion was determined by comparison of the methine proton of the ketone at 2.59 ppm (1H) with the methine proton of the secondary alcohol at 1.59 ppm in the ^{1}H-NMR spectrum.

The ATH of other ketones with SCM **3** was carried out using the same procedure as for acp and the products were analyzed by comparing their chiral HPLC and ^{1}H-NMR data with the literature.[4-9]

3.2 Analytic data of ATH products

While all the products we tried have previously been reported in the literature,[5, 10-18] the analytic details of some sample products are given below.

(*R*)-4-(1-Hydroxyethyl)benzoic acid

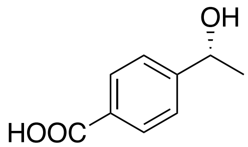

(Adjust the aqueous solution to pH 7)

Chiral HPLC (i-PrOH/hexane 20:80, 1.0 mL/min flow rate. Column: Daicel Chiralcel OM, wavelength = 230 nm): 11.50 min (R); 6.30 min (S).

^{1}H NMR (400 MHz, $CDCl_3$) δ 8.16 (d, J = 8.7 Hz, 2H), 8.02 (d, J = 8.6 Hz, 2H), 1.50 (d, J = 6.4 Hz, 3H).

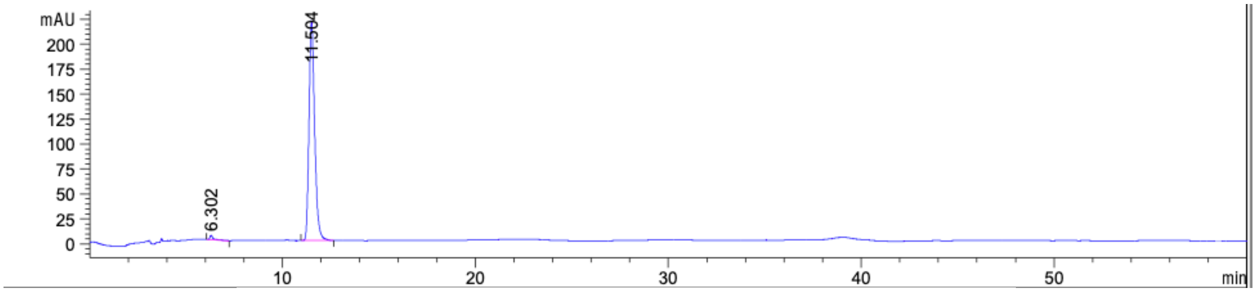

Signal 3: DAD1 D, Sig=230,4 Ref=360,100

Peak #	RetTime [min]	Type	Width [min]	Area [mAU*s]	Height [mAU]	Area %
1	6.302	BB	0.1725	54.90618	4.43234	1.2134
2	11.504	BB	0.3054	4470.12354	219.66136	98.7866
Totals :				4525.02971	224.09370	

Figure S15. Chiral HPLC spectrum of (*R*)-4-(1-Hydroxyethyl)benzoic acid.

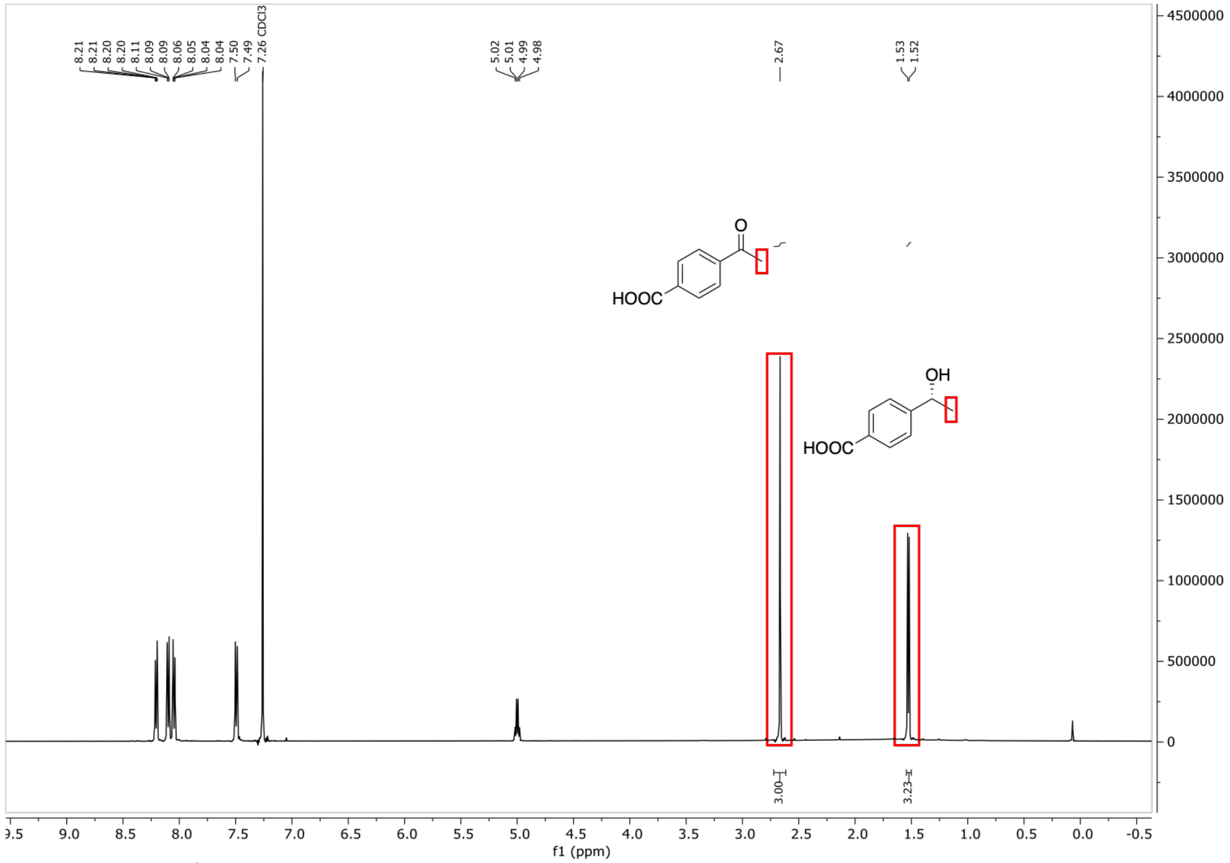

Figure S16. ^{1}H-NMR spectrum of the mixture of 4-acetylbenzoic acid and (*R*)-4-(1-hydroxyethyl)benzoic acid. ATH reaction on 4-acetylbenzoic acid at 60 °C under visible light using SCM **3** as catalyst after 48 hours.

(*R*)-1-(3′-Pyridyl)ethanol

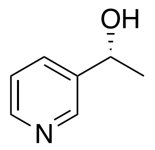

Chiral HPLC (i-PrOH/hexane 5:95, 0.5 mL/min flow rate. Column: Daicel Chiralcel OJ-H, wavelength = 254 nm): 34.50 min (R); 28.17 min (S).

^{1}H NMR (400 MHz, $CDCl_3$) δ 8.61 (d, J = 2.0Hz, 1H), 8.52 (dd, J = 4.8Hz, 3.2Hz, 1H), 7.75-7.73 (m, 1H), 7.31-7.26 (m, 1H), 4.96 (q, J = 6.4Hz, 1H), 1.53 (d, J = 6.4Hz, 3H).

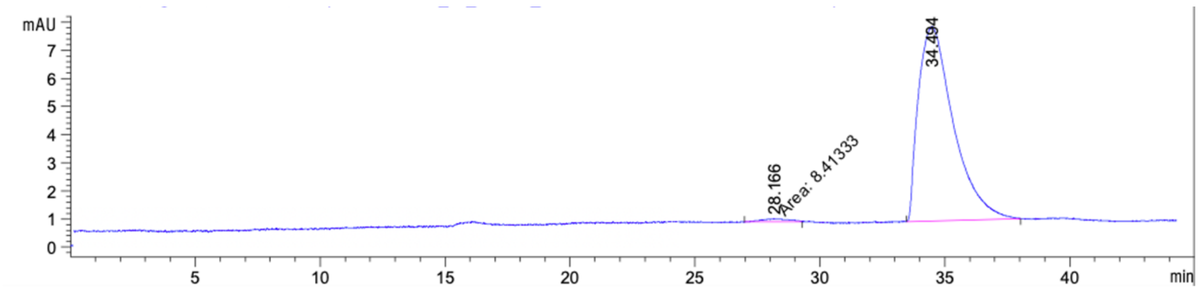

Signal 1: DAD1 B, Sig=254,4 Ref=360,100

Peak #	RetTime [min]	Type	Width [min]	Area [mAU*s]	Height [mAU]	Area %
1	13.467	BB	0.3293	36.19099	1.57964	4.9829
2	23.851	BB	0.6530	690.11157	16.07172	95.0171
Totals :				726.30256	17.65136	

Figure S17. Chiral HPLC spectrum of (*R*)-1-(3′-Pyridyl)ethanol.

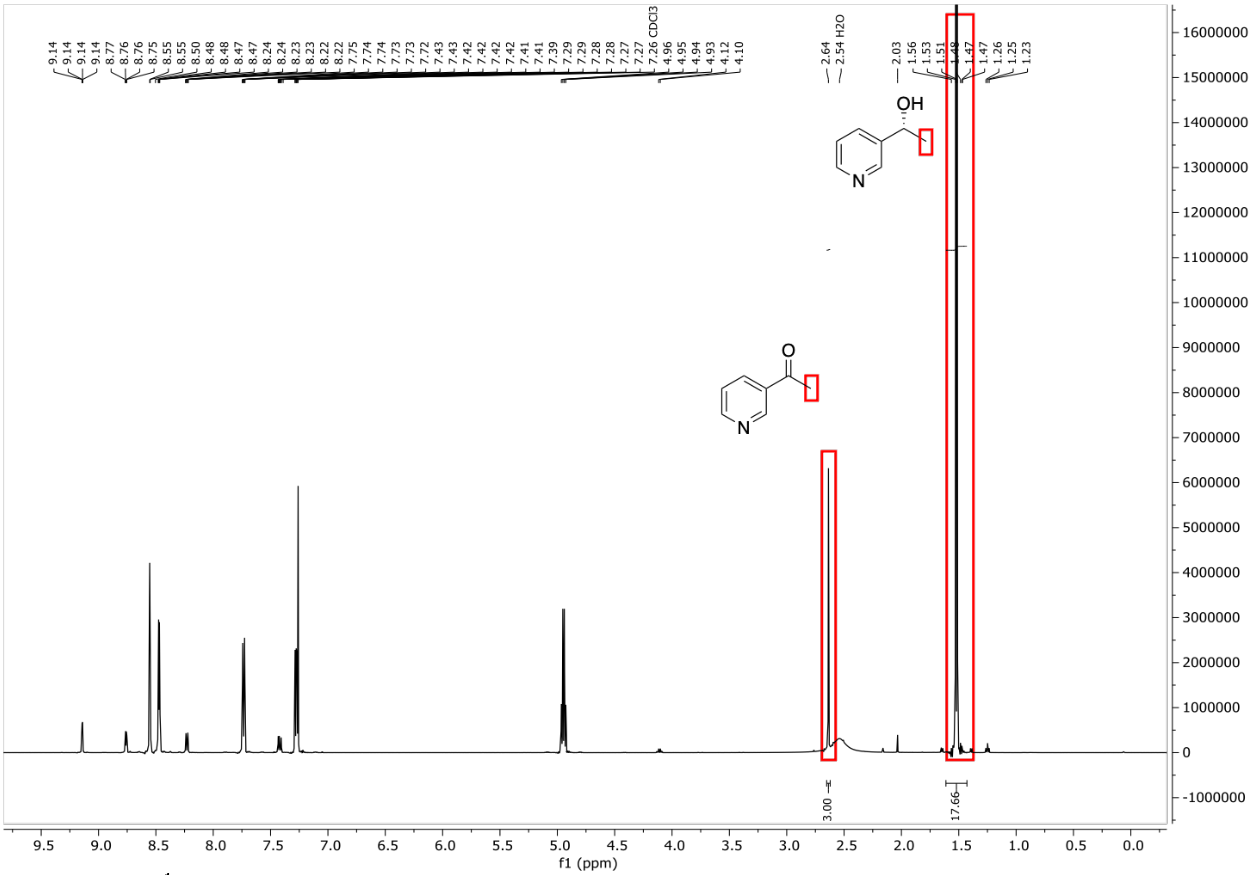

Figure S18. ^{1}H-NMR spectrum of the mixture of 3-acetylpyridine and *(R)*-1-(3-pyridyl)ethanol. ATH reaction on 3-acetylpyridine at 60 °C under UV light using SCM **3** as catalyst after 48 hours.

(*R*)-4-(1-Hydroxyethyl)phenol

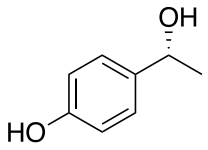

Chiral HPLC (i-PrOH/hexane 3:97, 1 mL/min flow rate. Column: Daicel Chiralcel OM, wavelength = 210 nm): 19.26 min (R); 17.46 min (S).

^{1}H NMR (600 MHz, $CDCl_3$) δ 7.25 (d, *J* = 8.6 Hz, 2H), 6.82 (d, *J* = 8.5 Hz, 2H), 4.87 (q, *J* = 12 Hz, 1H), 1.48 (d, *J* = 12 Hz, 3H).

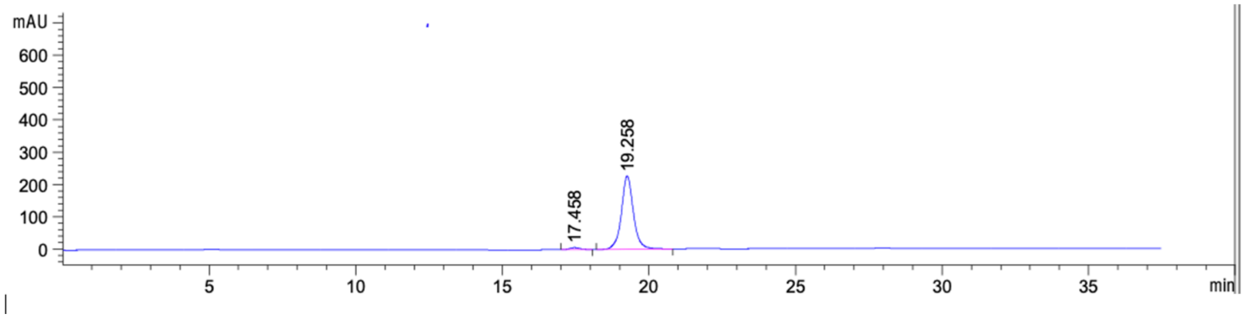

Signal 3: DAD1 C, Sig=210,4 Ref=360,100

Peak #	RetTime [min]	Type	Width [min]	Area [mAU*s]	Height [mAU]	Area %
1	17.458	BB	0.3342	134.72578	5.90565	2.0521
2	19.258	BB	0.4250	6430.51611	226.62755	97.9479

Totals : 6565.24190 232.53320

Figure S19. Chiral HPLC spectrum of (*R*)-4-(1-Hydroxyethyl)phenol.

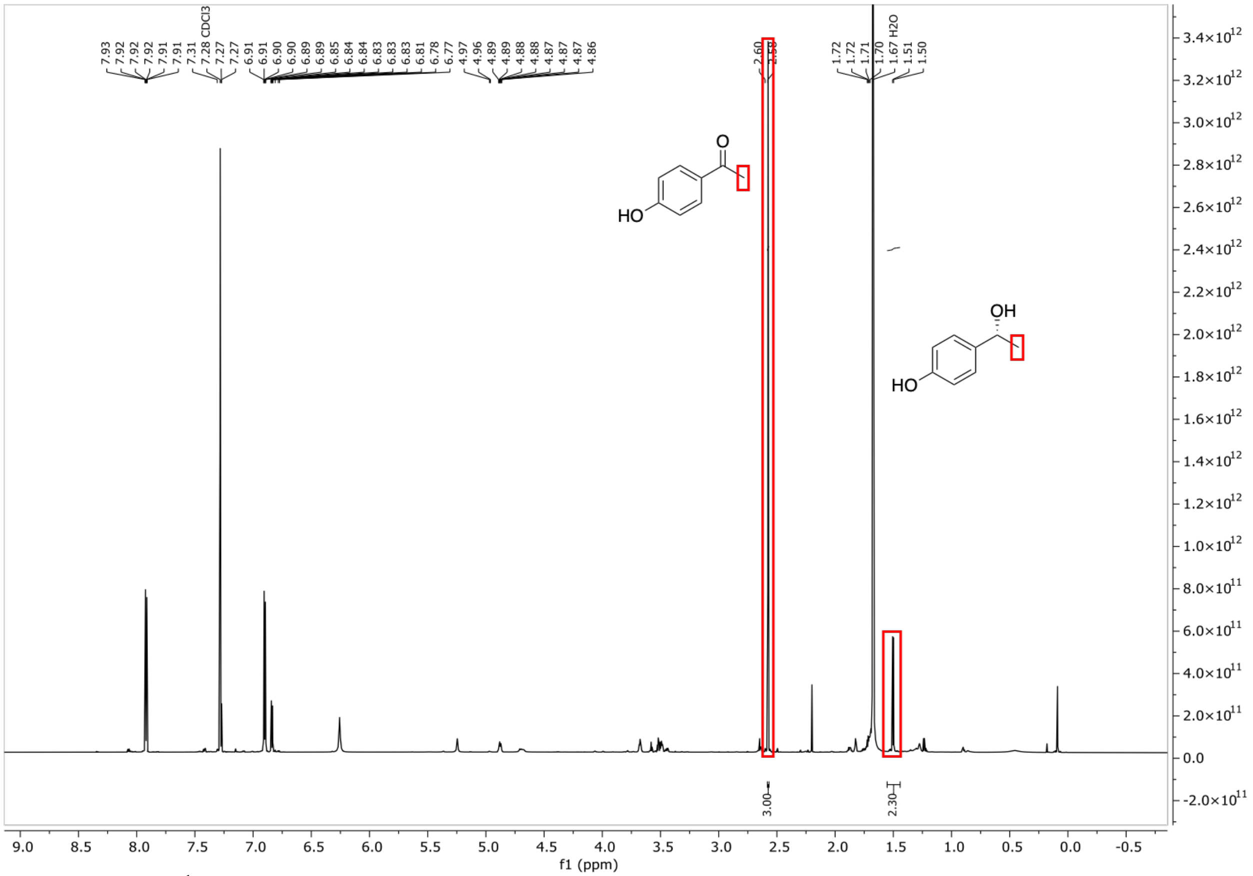

Figure S20. ^{1}H-NMR spectrum of the mixture of 4'-hydroxyacetophenone and (*R*)-4-(1-hydroxyethyl)phenol. ATH reaction on 4'-hydroxyacetophenone at 40 °C under visible light using SCM **3** as catalyst after 48 hours.

(*R*)-1-(4-Methoxyphenyl)ethanol

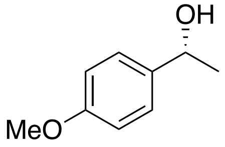

Chiral HPLC (i-PrOH/hexane 1:99, 1.0 mL/min flow rate. Column: Daicel Chiralcel OD-H, wavelength = 254 nm): 11.35 min (R); 14.75 min (S).

^{1}H NMR (400 MHz, $CDCl_3$) δ 7.33-7.28 (m, 2H), 6.91-6.85 (m, 2H), 4.86 (q, J = 6.4 Hz, 1H), 3.81 (s, 3H), 1.76 (s, 1H), 1.49 (d, J = 6.4 Hz, 3H).

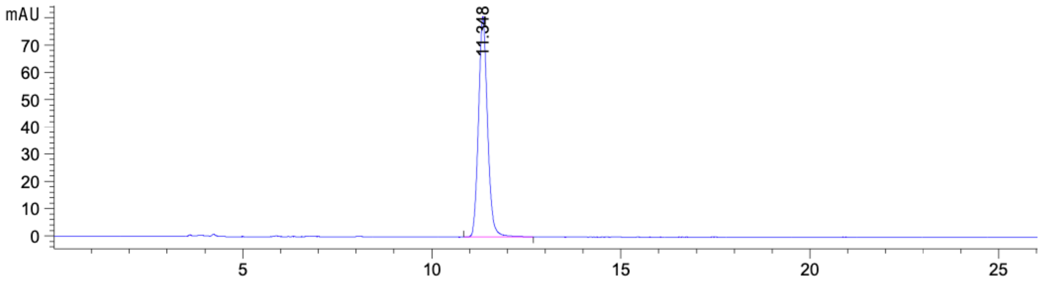

Signal 2: DAD1 B, Sig=254,4 Ref=360,100

Peak #	RetTime [min]	Type	Width [min]	Area [mAU*s]	Height [mAU]	Area %
1	11.348	BB	0.2544	1347.33374	80.85840	100.0000
Totals :				1347.33374	80.85840	

Figure S21. Chiral HPLC spectrum of (*R*)-1-(4-methoxyphenyl)ethanol.

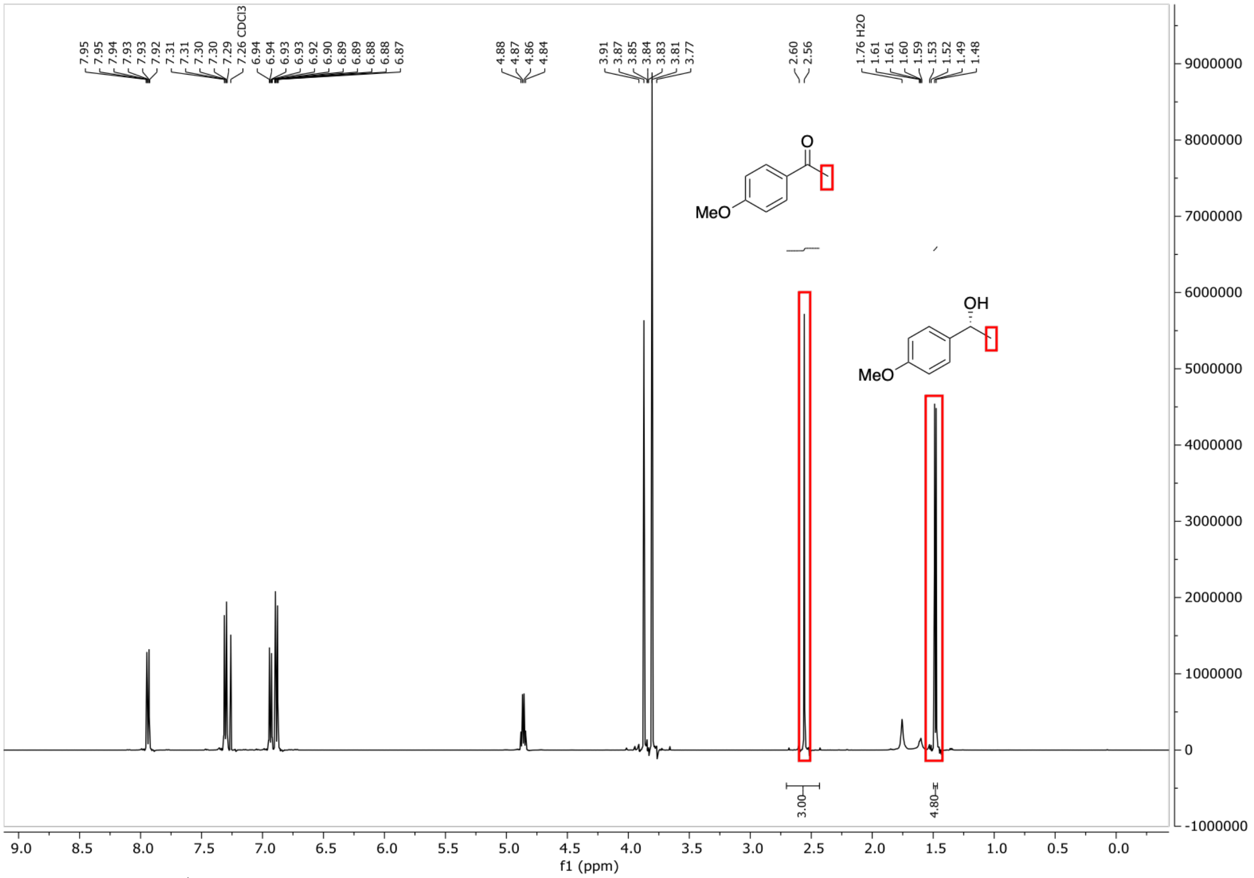

Figure S22. ^{1}H-NMR spectrum of the mixture of 4'-methoxyacetophenone and (*R*)-1-(4-methoxyphenyl)ethanol. ATH reaction on 4'-methoxyacetophenone at 60 °C under visible light using SCM **3** as catalyst after 48 hours.

(*R*)-1-(4-Nitrophenyl)ethanol

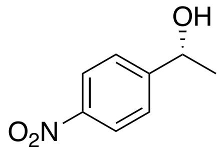

Chiral HPLC (i-PrOH/hexane 10:90, 0.5 mL/min flow rate. Column: Daicel Chiralcel OD-H, wavelength = 254 nm): 21.88 min (R); 16.86 min (S).

^{1}H NMR (400 MHz, $CDCl_3$) δ 8.01 (d, J = 8.5 Hz, 2H,), 7.80 (d, J = 8.0 Hz, 2H,), 4.95 (q, J = 6.5 Hz, 1H,), 1.52 (d, J = 6.5 Hz, 3H).

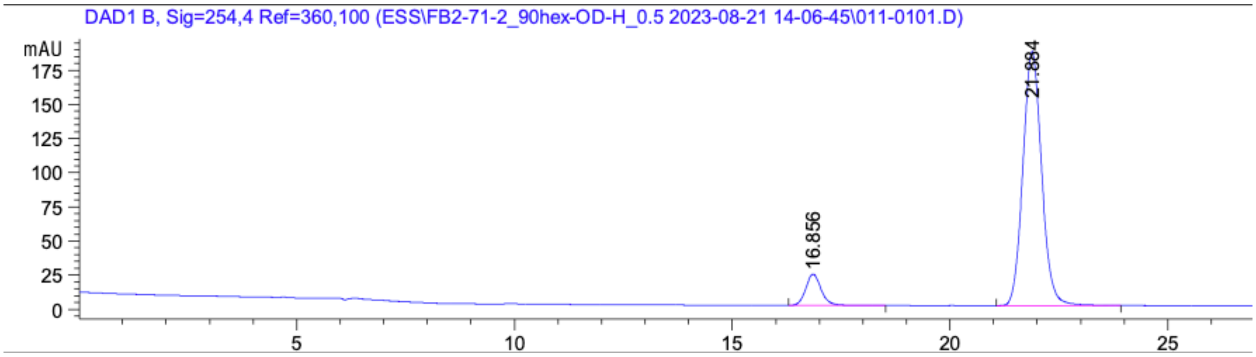

Signal 1: DAD1 B, Sig=254,4 Ref=360,100

Peak #	RetTime [min]	Type	Width [min]	Area [mAU*s]	Height [mAU]	Area %
1	16.856	BB	0.3812	563.59021	22.89387	9.1897
2	21.884	BB	0.4610	5569.26318	186.20026	90.8103
Totals :				6132.85339	209.09413	

Figure S23. Chiral HPLC spectrum of (*R*)-1-(4-nitrophenyl)ethanol.

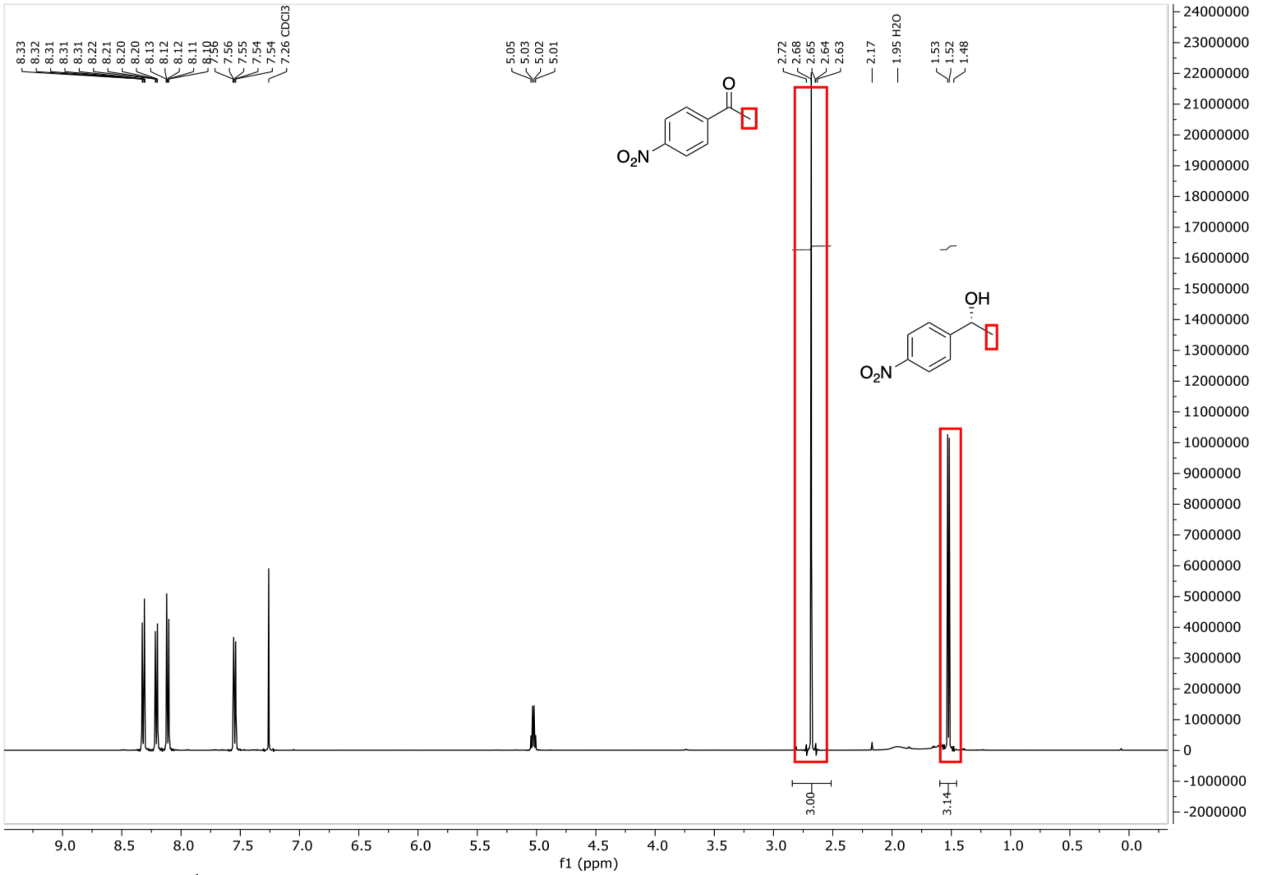

Figure S24. ^{1}H-NMR spectrum of the mixture of 4'-nitroacetophenone and (*R*)-1-(4-Nitrophenyl)ethanol. ATH reaction on 4'-nitroacetophenone at 40 °C under visible light using SCM **3** as catalyst after 48 hours.

(*R*)-1-Phenylethanol

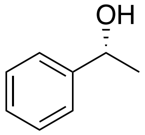

Chiral HPLC (i-PrOH/hexane 5:95, 0.4 mL/min flow rate. Column: Daicel Chiralcel OD-H, wavelength = 210 nm): 19.442 min (R); 23.934 min (S).

^{1}H NMR (400 MHz, $CDCl_3$) δ 7.32–7.41 (m, 3 H), 7.24–7.28 (m, 2H), 4.91 (q, *J* = 6.5 Hz, 1H), 1.86 (brs, 1H), 1.50 ppm (d, *J* = 6.5Hz, 3H).

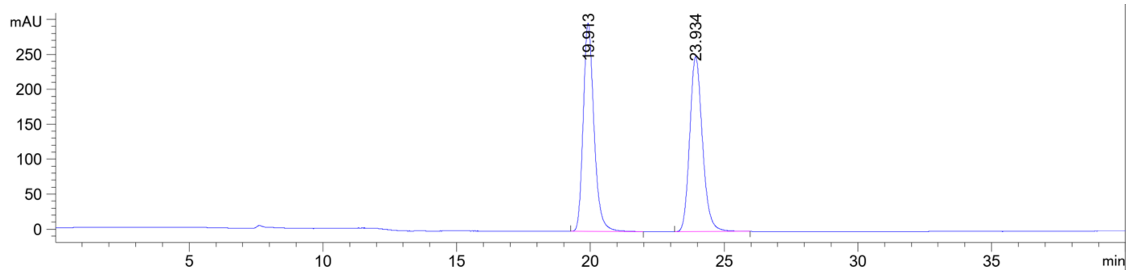

Signal 3: DAD1 C, Sig=210,4 Ref=360,100

Peak #	RetTime [min]	Type	Width [min]	Area [mAU*s]	Height [mAU]	Area %
1	19.913	BB	0.4288	8347.50781	297.98645	50.1132
2	23.934	BB	0.5114	8309.80664	250.26289	49.8868
Totals :				1.66573e4	548.24934	

Figure S25. Chiral HPLC spectrum of racemic 1-phenylethanol.

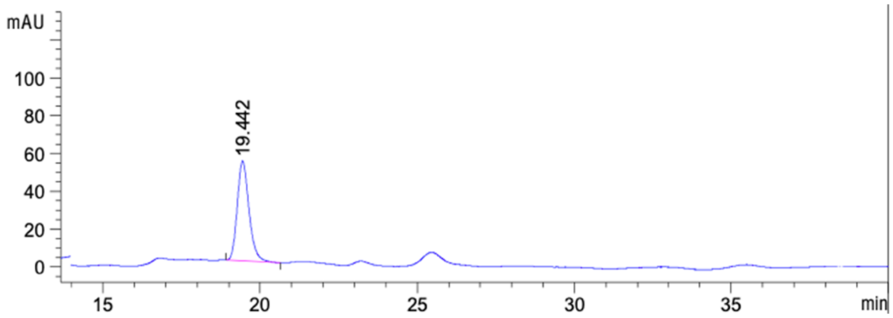

Signal 3: DAD1 C, Sig=210,4 Ref=360,100

Peak #	RetTime [min]	Type	Width [min]	Area [mAU*s]	Height [mAU]	Area %
1	19.442	BB	0.3998	1365.02112	52.77190	100.0000
Totals :				1365.02112	52.77190	

Figure S26. Chiral HPLC spectrum of (*R*)-1-phenylethanol.

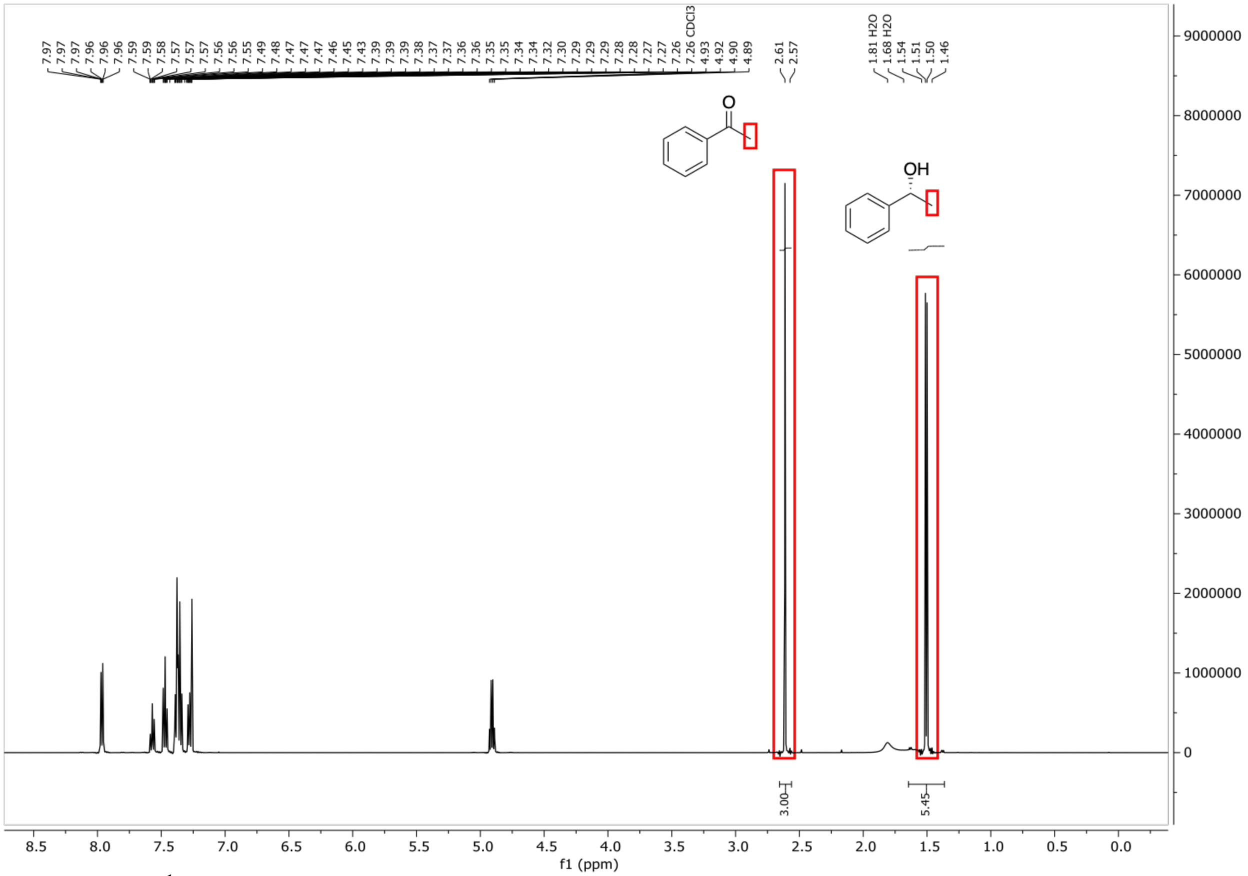

Figure S27. ^{1}H-NMR spectrum of the mixture of acetophenone and (*R*)-1-phenylethanol. ATH reaction on acetophenone at 60 °C under visible light using SCM **3** as catalyst after 48 hours.

(*R*)-1-(2'-Naphthyl)ethanol

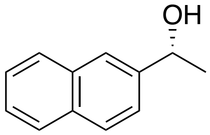

Chiral HPLC (i-PrOH/hexane 2:98, 1.0 mL/min flow rate. Column: Daicel Chiralcel OD-H, wavelength = 230 nm): 34.021 min (R); 32.141 min (S).

^{1}H NMR (400 MHz, $CDCl_3$) δ 7.25–7.83 (m, 7 H), 5.06 (q, *J*=6.5 Hz, 1 H), 1.89 (brs, 1H), 1.57 ppm (d, *J* = 6.5 Hz, 3H).

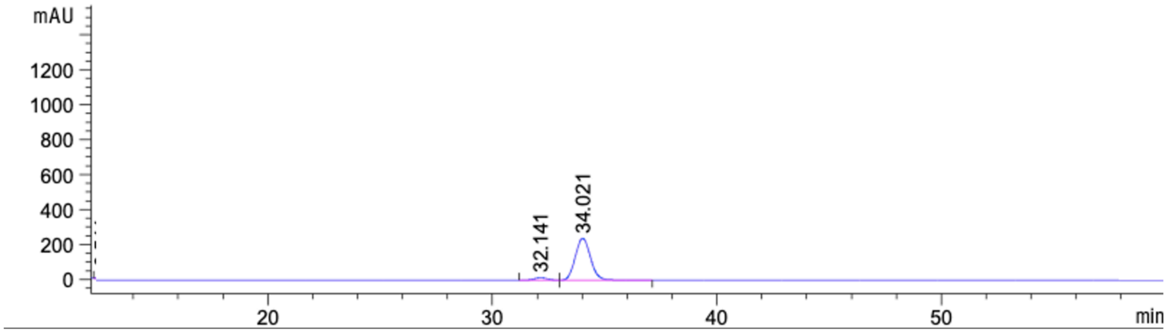

Signal 3: DAD1 D, Sig=230,4 Ref=360,100

Peak #	RetTime [min]	Type	Width [min]	Area [mAU*s]	Height [mAU]	Area %
1	32.141	BB	0.6007	546.43536	14.08221	4.6298
2	34.021	BB	0.7293	1.12562e4	239.94572	95.3702

Figure S28. Chiral HPLC spectrum of (*R*)-1-(2'-naphthyl)ethanol.

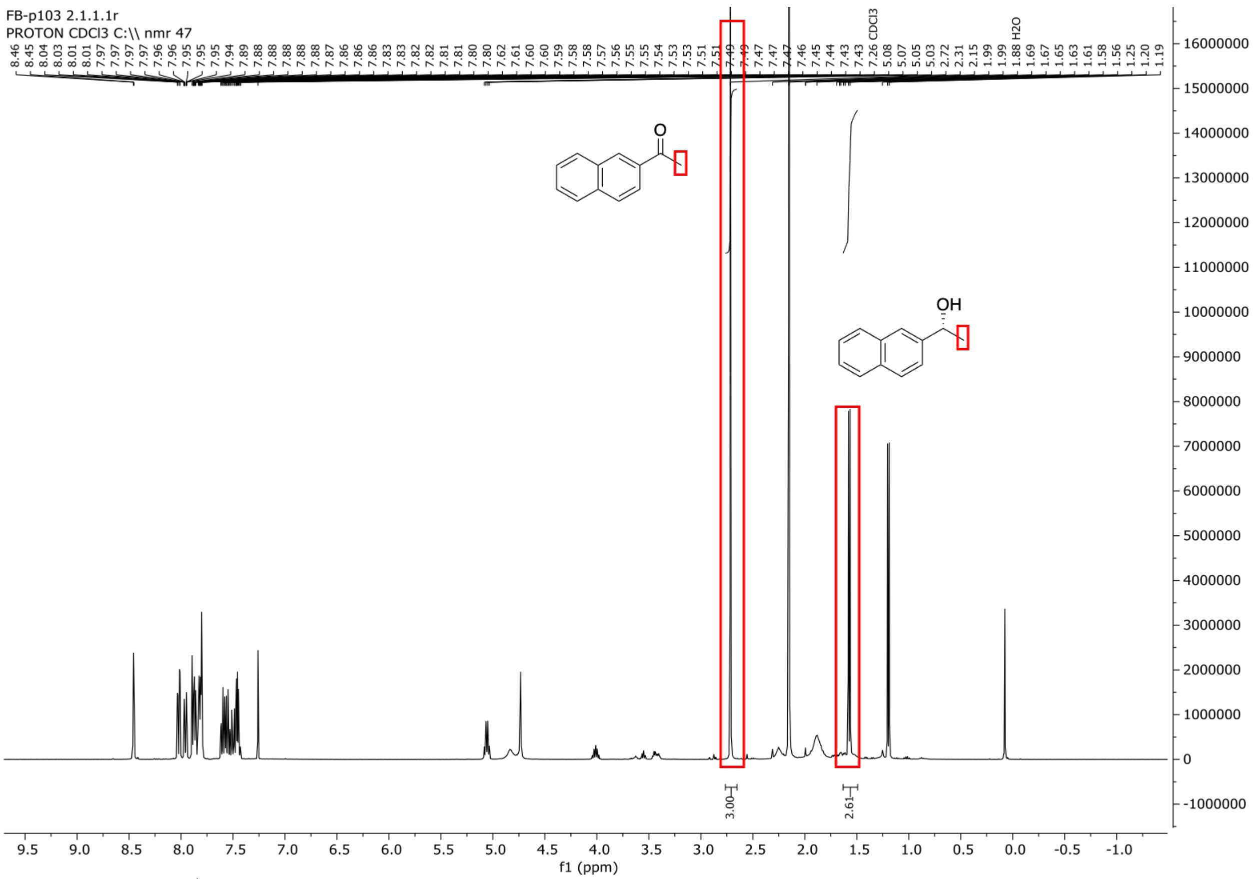

Figure S29. ^{1}H-NMR spectrum of the mixture of 2-acetylnaphthalene and (*R*)-1-(2'-naphthyl)ethanol. ATH reaction on 2-acetylnaphthalene at 60 °C under visible light using SCM **3** as catalyst after 48 hours.

(*R*)-1,2,3,4-Tetrahydronaphthalen-1-ol

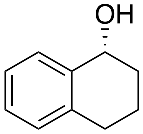

Chiral HPLC (i-PrOH/hexane 2:98, 1.0 mL/min flow rate. Column: Daicel Chiralcel OD-H, wavelength = 210 nm): 11.22 min (R); 9.96 min (S).

^{1}H NMR (400 MHz, $CDCl_3$) δ 7.47–7.39 (m, 1H), 7.24–7.18 (m, 2H), 7.13–7.09 (m, 1H), 4.78 (t, *J* = 5.0 Hz, 1H), 2.84 (dt, *J* = 16.6, 5.6 Hz, 1H), 2.73 (ddd, *J* = 16.6, 8.2, 5.7 Hz, 1H), 2.04 – 1.94 (m, 2H), 1.91 (dtd, *J* = 13.3, 7.2, 6.6, 3.7 Hz, 1H), 1.87 (s, 1H), 1.83–1.72 (m, 1H) ppm.

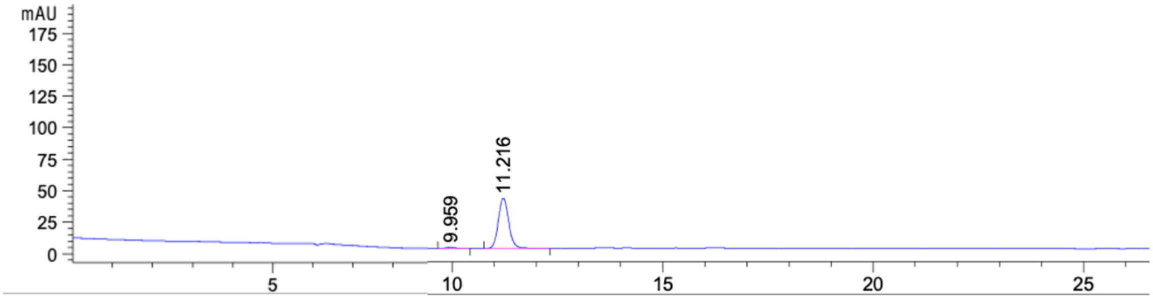

Signal 2: DAD1 C, Sig=210,4 Ref=360,100

Peak #	RetTime [min]	Type	Width [min]	Area [mAU*s]	Height [mAU]	Area %
1	9.959	BB	0.2185	67.96994	4.82103	1.9131
2	11.216	BB	0.2632	3484.84302	204.13609	98.0869

Totals : 3552.81296 208.95712

Figure S30. Chiral HPLC spectrum of (*R*)-1,2,3,4-tetrahydronaphthalen-1-ol.

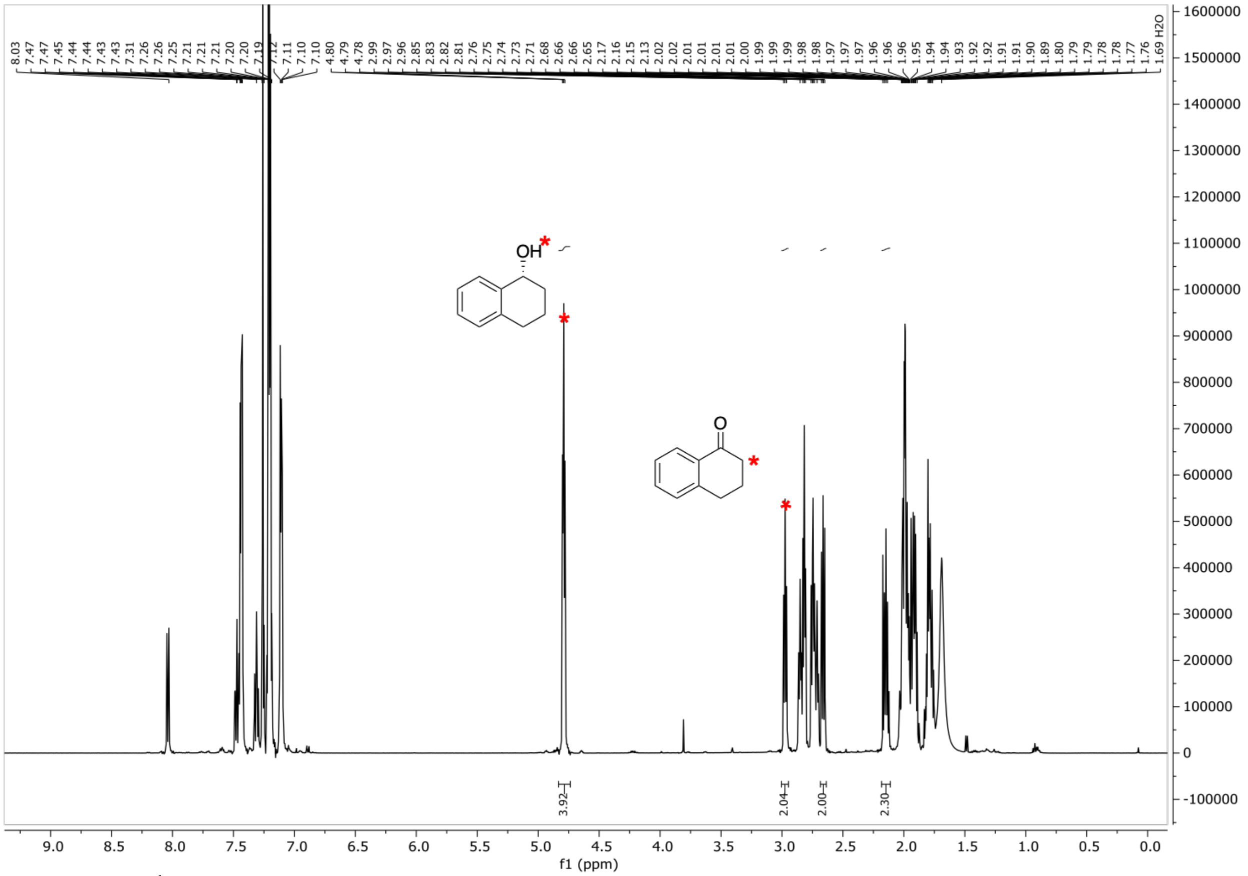

Figure S31. ^{1}H-NMR spectrum of the mixture of 1-tetralone and (*R*)-1,2,3,4-tetrahydronaphthalen-1-ol. ATH reaction on 1-Tetralone at 60 °C under UV light using SCM **3** as catalyst after 48 hours.

(*R*)-1-(Anthracen-2-yl)ethanol

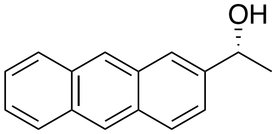

Chiral HPLC (i-PrOH/hexane 10:90, 0.8 mL/min flow rate. Column: Daicel Chiralcel OD-H, wavelength = 254 nm): 23.85 min (R); 13.47 min (S).

^{1}H NMR (400 MHz, $CDCl_3$) δ 8.27-8.32 (m, 5 H), 7.79-7.85 (m, 3 H), 5.09 (q, *J* = 6.5 Hz, 1 H), 1.61 ppm (d, *J* = 6.5 Hz, 3 H).

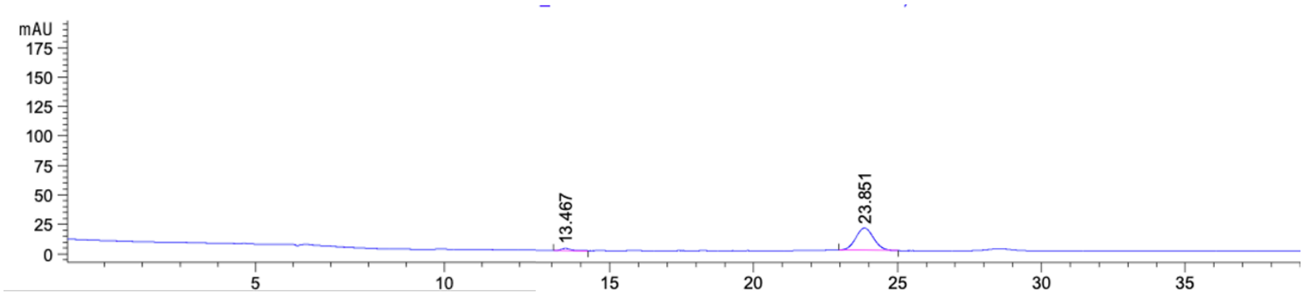

Signal 1: DAD1 B, Sig=254,4 Ref=360,100

Peak #	RetTime [min]	Type	Width [min]	Area [mAU*s]	Height [mAU]	Area %
1	13.467	BB	0.3293	36.19099	1.57964	4.9829
2	23.851	BB	0.6530	690.11157	16.07172	95.0171
Totals :				726.30256	17.65136	

Figure S32. Chiral HPLC spectrum of (*R*)-1-(Anthracen-2-yl)ethanol.

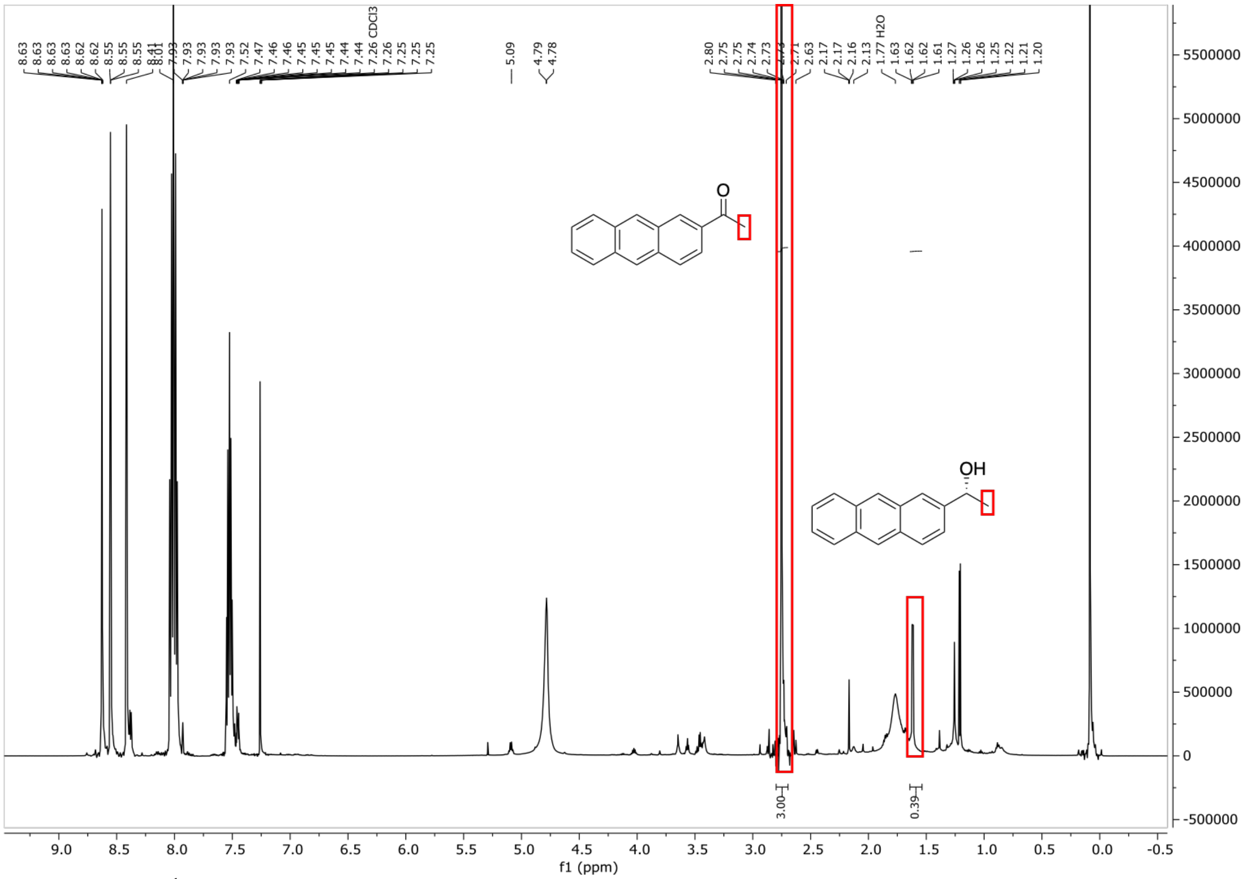

Figure S33. ^{1}H-NMR spectrum of the mixture of 2-acetylanthracene and (*R*)-1-(anthracen-2-yl)ethanol. ATH reaction on 2-acetylanthracene at 40 °C under visible light using SCM **3** as catalyst after 48 hours.

(1*R*)-1,3-Diphenyl-1-butanol

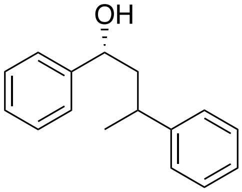

Chiral HPLC (i-PrOH/hexane 2:98, 1.0 mL/min flow rate. Column: Daicel Chiralcel OD-H, wavelength = 210 nm): 6.69 min (R); 9.72 min (S).

^{1}H NMR (400 MHz, $CDCl_3$) δ 7.33–7.29 (m, 4H), 7.25–7.20 (m, 6H), 4.43–4.41 (m, 1H), 3.05–3.01 (m, 1H), 2.06–2.01 (m, 1H), 1.97–1.93 (m, 1H), 1.28 (d, J = 6.8 Hz, 3H).

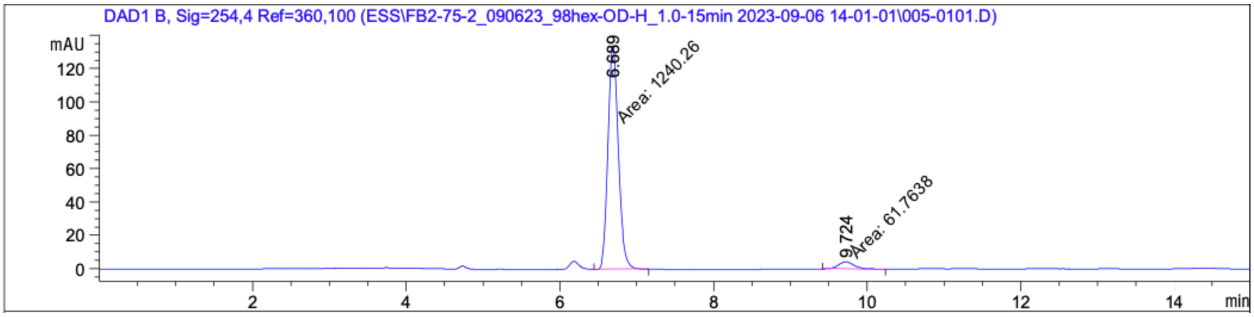

Signal 2: DAD1 B, Sig=254,4 Ref=360,100

Peak #	RetTime [min]	Type	Width [min]	Area [mAU*s]	Height [mAU]	Area %
1	6.689	MM	0.1543	1240.26404	134.00833	95.2563
2	9.724	MM	0.2550	61.76382	4.03608	4.7437

Totals : 1302.02786 138.04441

Figure S34. Chiral HPLC spectrum of (1*R*)-1,3-diphenyl-1-butanol.

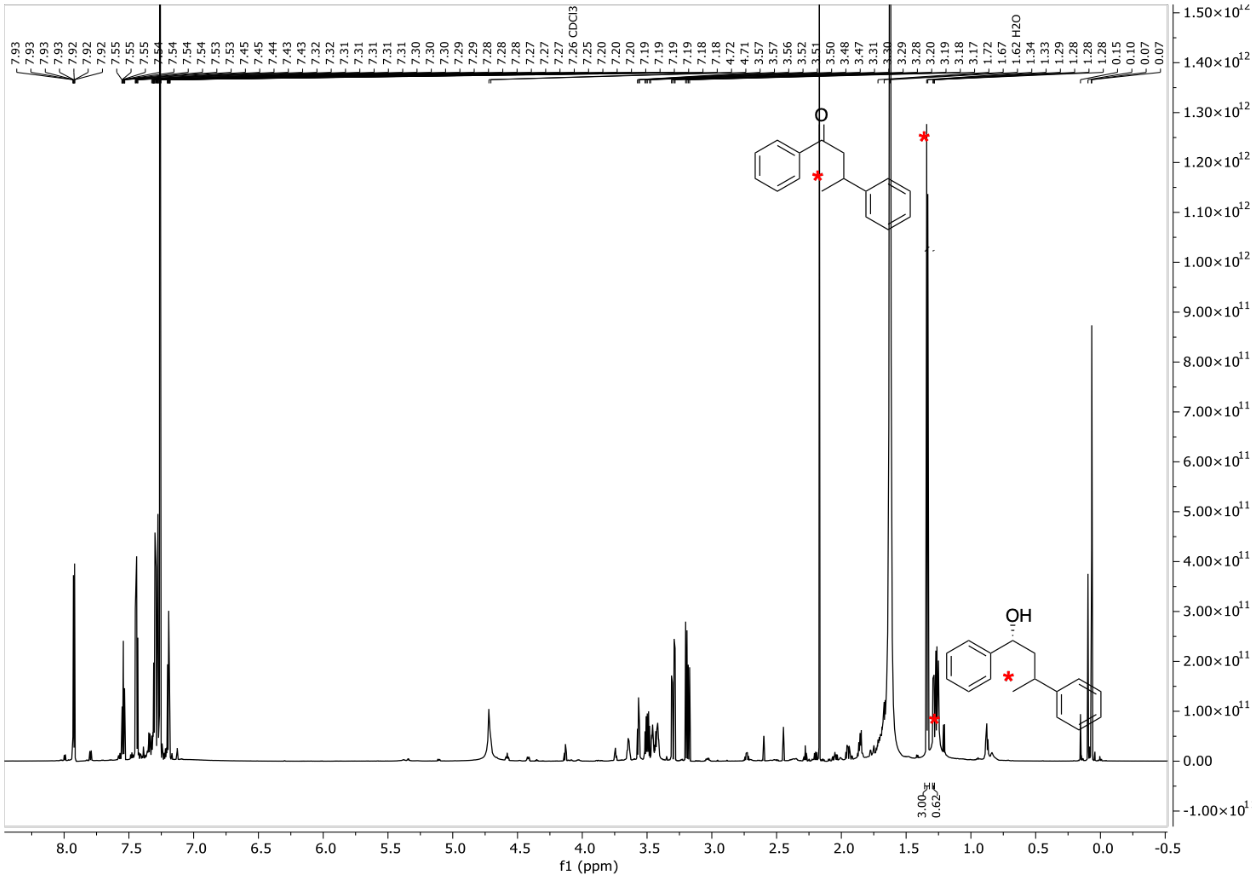

Figure S35. ^{1}H-NMR spectrum of the mixture of 1,3-diphenyl-1-butanone and (1*R*)-1,3-diphenyl-1-butanol. ATH reaction on 2-acetylanthracene at 40 °C under visible light using SCM **3** as catalyst after 48 hours.

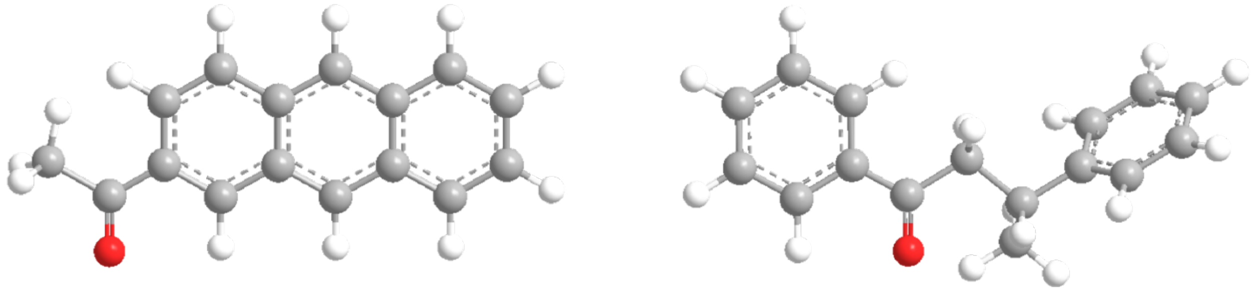

Figure S36. 3D models of 2-acetylanthracene (left) and 1,3-diphenyl-1-butanone (right).

4. References

1. Bodner, T.; Ellmaier, L.; Schenk, V.; Albering, J.; Wiesbrock, F., Delocalized π-electrons in 2-oxazoline rings resulting in negatively charged nitrogen atoms: revealing the selectivity during the initiation of cationic ring-opening polymerizations. *Polym. Int.* **2011,** *60*, 1173-1179.

2. Lu, J.; Liang, L.; Weck, M., Micelle-based nanoreactors containing Ru-porphyrin for the epoxidation of terminal olefins in water. *J. Mol. Cataly. A-Chem.* **2016,** *417*, 122-125.

3. Kuepfert, M.; Cohen, A. E.; Cullen, O.; Weck, M., Shell Cross-Linked Micelles as Nanoreactors for Enantioselective Three-Step Tandem Catalysis. *Chem. – Eur. J.* **2018,** *24*, 18648-18652.

4. Qu, P.; Kuepfert, M.; Hashmi, M.; Weck, M., Compartmentalization and Photoregulating Pathways for Incompatible Tandem Catalysis. *J. Am. Chem. Soc.* **2021,** *143*, 4705-4713.

5. Wu, X.; Li, X.; Zanotti-Gerosa, A.; Pettman, A.; Liu, J.; Mills, A. J.; Xiao, J., Rh III- and Ir III-catalyzed asymmetric transfer hydrogenation of ketones in water. *Chemistry* **2008,** *14*, 2209-22.

6. Zhang, G.; Han, X.; Luan, Y.; Wang, Y.; Wen, X.; Ding, C., L-Proline: an efficient N,O-bidentate ligand for copper-catalyzed aerobic oxidation of primary and secondary benzylic alcohols at room temperature. *Chem. Commun.* **2013,** *49*, 7908-10.

7. Gatenyo, J.; Vints, I.; Rozen, S., The first general route for efficient synthesis of 18O labelled alcohols using the HOF.CH3CN complex. *Chem. Commun.* **2013,** *49*, 7379-81.

8. Zhang, G.; Cheng, J.; Davis, K.; Bonifacio, M. G.; Zajaczkowski, C., Practical and selective hydroboration of aldehydes and ketones in air catalysed by an iron(II) coordination polymer. *Green Chem.* **2019,** *21*, 1114-1121.

9. Deng, D.; Meng, Q.; Li, Z.; Ma, R.; Yang, Y.; Wang, Z.; Zhang, N.; Zou, X.; Zhu, G.; Yuan, Y., Enzyme-Inspired Assembly: Incorporating Multivariate Interactions to Optimize the Host-Guest Configuration for High-Speed Enantioselective Catalysis. *ACS Appl. Mater. Interfaces* **2020,** *12*, 47966-47974.

10. Yu, J.; Long, J.; Yang, Y.; Wu, W.; Xue, P.; Chung, L. W.; Dong, X.-Q.; Zhang, X., Iridium-catalyzed asymmetric hydrogenation of ketones with accessible and modular ferrocene-based amino-phosphine acid (f-ampha) ligands. *Org. Lett.* **2017,** *19*, 690-693.

11. Neupert, A.; Ress, T.; Wittmann, J.; Hummel, W.; Gröger, H., Enantioselective Biocatalytic Reduction of Non-protected Hydroxyacetophenones. *Z. Naturforsch. B.* **2010,** *65*, 337-340.

12. Abazid, A. H.; Clamor, N.; Nachtsheim, B. J., An Enantioconvergent Benzylic Hydroxylation Using a Chiral Aryl Iodide in a Dual Activation Mode. *ACS Catal.* **2020,** *10*, 8042-8048.

13. Kodama, K.; Kobayashi, Y.; Saigo, K., Two-component supramolecular helical architectures: creation of tunable dissymmetric cavities for the inclusion and chiral recognition of the third components. *Chemistry* **2007,** *13*, 2144-52.

14. Contente, M. L.; Serra, I.; Palazzolo, L.; Parravicini, C.; Gianazza, E.; Eberini, I.; Pinto, A.; Guidi, B.; Molinari, F.; Romano, D., Enzymatic reduction of acetophenone derivatives with a benzil reductase from Pichia glucozyma (KRED1-Pglu): electronic and steric effects on activity and enantioselectivity. *Org. Biomol. Chem.* **2016,** *14*, 3404-3408.

15. Liu, F.; Qu, P.; Weck, M., Photoresponsive Azobenzene-Functionalized Shell Cross-Linked Micelles for Selective Asymmetric Transfer Hydrogenation. *Org. Lett.* **2022,** *24*, 4099-4103.

16. Ito, J.-i.; Ujiie, S.; Nishiyama, H., New Bis(oxazolinyl)phenyl−Ruthenium(II) Complexes and Their Catalytic Activity for Enantioselective Hydrogenation and Transfer Hydrogenation of Ketones. *Organometallics* **2009,** *28*, 630-638.

17. Li, Y.; Yu, S.; Wu, X.; Xiao, J.; Shen, W.; Dong, Z.; Gao, J., Iron Catalyzed Asymmetric Hydrogenation of Ketones. *J. Am. Chem. Soc.* **2014,** *136*, 4031-4039.

18. Yang, Y.; Guo, J.; Ng, H.; Chen, Z.; Teo, P., Formal hydration of non-activated terminal olefins using tandem catalysts. *Chem. Commun.* **2014,** *50*, 2608-2611.
